# Synthesis and Functionalization of Periodic Copolymers

**DOI:** 10.3390/polym9050166

**Published:** 2017-05-06

**Authors:** Falk Kubatzki, Lucas Al-Shok, Niels ten Brummelhuis

**Affiliations:** Department of Chemistry, Humboldt-Universität zu Berlin, Brook-Taylor-Str. 2, Berlin 12489, Germany; falk.kubatzki@chemie.hu-berlin.de (F.K.); Lucas.Al-Shok@gmx.net (L.A.-S.)

**Keywords:** alternating copolymer, periodic copolymer, sequence control, post-polymerization functionalization

## Abstract

For the copolymerization of non-conjugated olefins and maleimides, it is known that under certain conditions periodic ABA monomer sequences are formed. In this work, such a copolymerization is used to create polymers which have defined (periodic) monomer sequences and can be functionalized after polymerization. The copolymerization of pentafluorophenol (PFP) active esters of 4-pentenoic acid and perillic acid with *N*-phenyl maleimide (PhMI) was studied in 1,2-dichloroethane (DCE) and 1,1,1,3,3,3-hexafluoro-2-phenyl-2-propanol (HFPP). In DCE and for the copolymerization of the PFP ester of 4-pentenoic acid and PhMI in HFPP, polymers were formed where the active esters were separated by at least one PhMI unit. The average number of separating PhMI units can be controlled by varying the feed ratio of the monomers. For the copolymerization of the PFP ester of perillic acid in HFPP, a preference for the formation of periodic copolymers was observed, where active esters were preferably separated from each other by a maximum of two PhMI moieties. Therefore, the copolymerization of said active ester containing monomers with PhMI provides a platform to create polymers in which reactive moieties are distributed along the polymer chain in different fashions. The active esters in the non-conjugated vinyl monomers could be used in a post-polymerization functionalization step to create functionalized polymers with defined monomer sequences in a modular way.

## 1. Introduction

It is well known that the structure of macromolecules strongly relates to their properties [1,2,3,4,5]. Structural features such as the architecture, molecular weight (or distribution thereof), tacticity and monomer sequence all play a role. Especially in chain-growth polymerizations, controlling these properties can be challenging because of the reactive nature of the propagating species. Whereas significant progress has been made to control the architecture and molecular weight of synthetic polymers (notably using controlled free-radical techniques [6,7,8,9]) as well as the tacticity (e.g., using metal-catalyzed polymerization [10], but also in radical polymerizations [11]), the monomer sequence is still difficult to control.

Besides alternating copolymers, which can be prepared with relative ease, e.g., by choosing a pair of monomers that are electron-rich and -deficient, respectively [12,13,14,15], hardly any sequence-defined polymers are known for chain-growth polymerizations. Periodic ABA copolymers, the second simplest sequence imaginable, have e.g., only been described a few times in the literature.

One method of creating periodic copolymers is to use pre-organized monomers. Sawamoto and coworkers e.g., used a cyclopolymerization approach in which two covalently linked styrene derivatives and a third, supramolecularly bound, monomer (4-vinyl pyridine) were pre-organized into an ABA-assembly which, upon polymerization, could be incorporated into the polymer chain as a single unit [16]. Similarly, pre-organization of monomers can also be used to create other types of simple monomer sequences [17,18].

The (controlled) free-radical copolymerization of some (bulky) non-conjugated olefins with maleimides presents another approach for the preparation of periodic ABA copolymers. For such copolymerizations, it has been shown that ABA-type periodic copolymers can be formed [19,20,21,22] rather than the alternating copolymers that are typically found in the copolymerization of e-rich and -deficient monomers. This tendency towards the formation of ABA-periodic copolymers is most pronounced in Lewis acidic solvents, in particular, in a perfluorinated alcohol (1,1,1,3,3,3-hexafluoro-2-phenyl-2-propanol, HFPP), which might be due to a supramolecular pre-organization of the maleimide monomers, as well as by the further decrease of the electron-density in the already e-deficient vinyl group of the maleimide, thereby limiting the homopropagation of these monomers. Similar penultimate effects were observed for the copolymerization of e-rich styrene derivatives with maleic anhydride in 1,4-dioxane [23].

The severely limited number of systems in which periodic copolymers are found makes a post-polymerization functionalization approach highly interesting for the synthesis of functional periodic copolymers [12,24,25]. In such an approach, functionalizable monomers would be used in a periodic copolymerization. These reactive groups can, in a second step, be functionalized without disturbing the monomer sequence, thereby giving rise to polymers in which functional groups are distributed along the polymer chain in a highly defined manner. In this highly modular strategy, only one monomer pair is required to be able to prepare a wide variety of different, functional periodic copolymers.

To accomplish this goal, the copolymerization of two active ester-containing non-conjugated vinyl monomers (the pentafluorophenyl esters of 4-pentenoic acid and (S)-perillic acid, abbreviated as PentPFP and PerPFP respectively) with *N*-phenyl maleimide (PhMI) was investigated, in the hope of obtaining ABA-periodic copolymers, in which A represents the maleimide and B the monomer containing an active ester. After the polymerization, the active ester can be used to functionalize the copolymer with e.g., amine containing moieties [26,27].

## 2. Materials and Methods

### 2.1. Materials

The following chemicals were used as received, unless mentioned otherwise. Pentafluorophenol (Carbolution Chemicals GmbH, Saarbrücken, Germany, 99%), 4-pentenoic acid (Sigma-Aldrich, Steinheim, Germany, 95%), (S)-perillic acid (Sigma-Aldrich, Buchs, Switzerland, 95%), *N*-phenylmaleimide (Sigma-Aldrich, Steinheim, Germany, 97%), *N,N′*-dicyclohexylcarbodiimide (Sigma-Aldrich, Steinheim, Germany 99%), 4-dimethylaminopyridine (Sigma-Aldrich, Steinheim, Germany, 99%), 1,2-dichloroethan (Alfa Aesar, ThermoFisher (Kandel) GmbH, Karlsruhe, Germany, 99+%), 1,4-dioxane (Acros Organics, Geel, Belgium, 99.5%), *N,N*-dimethylformamide (Sigma-Aldrich, Steinheim, Germany, 99.8%), 2,2′-azobis (2-methylpropionitrile) (Acros Organics, Geel, Belgium, 98%), *n*-hexylamine (Sigma-Aldrich, Steinheim, Germany, 99%), benzylamine (MERCK, Kenilworth, NJ, USA, >99%), CDCl_3_ (Deutero GmbH, Kastellaun, Germnay, 99.8%), 1,1,1,3,3,3-hexafluoro-2-phenyl-2-propanol (abcr, Karlsruhe, Germany, 99%). 2,2′-Azobis (2-methylpropionitrile) (AIBN, Acros Organics, Geel, Belgium, 98%) was recrystallized from methanol.

### 2.2. Characterization Methods

#### 2.2.1. NMR Spectroscopy

NMR spectroscopic measurements were performed on a Bruker Avance III-300 spectrometer (operating at 300 MHz for ^1^H NMR and 75 MHz for ^13^C) and on a Bruker Avance III-500 spectrometer (operating at 500 MHz for ^1^H NMR and 126 MHz for ^13^C) from Bruker Biospin GmbH (Rheinstetten, Germany) at 20 °C. All chemical shifts (δ) are reported in ppm relative to solvent residual signals of the deuterated solvent.

#### 2.2.2. Size Exclusion Chromatography (SEC)

SEC was carried out on a WEG Dr. Bures-Systems (WEG Dr. Bures GmbH & Co. KG, Dallgow-Döberitz, Germany). Measurements were performed at a flow rate of 1.0 mL/min at 60 °C in THF. The column set consisted of three 300 × 8 mm SDV columns (50 Å 5 µm, 500 Å 5 µm, 1000 Å 5 µm) and one 50 × 8 mm SDV column from PSS Polymer Standards Service GmbH). Commercially available polystyrene (PS) standards were used for calibration.

#### 2.2.3. Microwave-Assisted Post-Polymerization Functionalization

Microwave-assisted post-polymerization functionalization experiments were carried out on a Discover SP-D Microwave by CEM GmbH (Kamp-Linfort, Germany).

#### 2.2.4. MALDI-TOF-MS

Matrix-Assisted Laser Desorption/Ionization Time-Of-Flight Mass Spectrometry (MALDI-TOF MS) measurements were performed on an Autoflex III Smartbeam system from Bruker (Billerica, MA, USA). A solution containing 7 mg of α-cyano-4-hydroxycinnamic acid or 7 mg of 2,5-dihydroxybenzoic acid in 1 mL of a 50:50:0.1 acetonitrile-water-trifluoroacetic acid solution was used as a matrix solution. 2 mg of the polymer was dissolved in 1 mL of a 50:50:0.1 acetonitrile-water-trifluoroacetic acid solution. 1 mL of this polymer solution was mixed with 1.5 mL of the matrix solution on the substrate; 1 mL of this solution was taken and diluted with another 1.5 mL of the matrix solution. The samples were dried at room temperature. The measurements were performed in positive mode.

#### 2.2.5. ESI-MS

Electron-Spray Ionization Mass Spectrometry (ESI-MS) measurements were performed on an Acquity-UPLC H-class CM Core system (Waters Corporation, Milford, CT, USA) with an Acquity-UPLC PDA and QDa mass detector and a LCT Premier XE mass spectrometer for Ultra Performance Liquid Chromatography-High Resolution Mass Spectrometry (UPLC-HRMS). The measurements were conducted using the bypass function with either 99.9% acetonitrile with 0.1% formic acid or with methanol as the solvent.

### 2.3. Monomer Synthesis

#### 2.3.1. 4-Pentenoic Acid Pentafluorophenyl Ester (PentPFP)

PentPFP was synthesized according to a slightly adapted literature procedure [28]. 4-Pentenoic acid (1.2 g, 12 mmol, 1 eq.) was dissolved in 1,4-dioxane (48 mL) under argon in a round-bottom flask. Pentafluorophenol (2.43 g, 13.2 mmol, 1.1 eq.), *N,N′*-dicyclohexylcarbodiimide (2.72 g, 13.2 mmol, 1.1 eq.) and 4-dimethylaminopyridine (146 mg, 1.2 mmol, 0.1 eq.) were added to the reactions mixture. The resulting white suspension was stirred for 16 h at rt. The precipitate was filtered off and washed with chloroform (10 mL). The combined organic phases were concentrated *in vacuo* and the residue was purified with column chromatography using 95:5 *v*/*v* hexane:ethyl acetate. The product was obtained as a colorless oil (2.27 g, 71%).

^1^H-NMR (500 MHz, CDCl_3_, δ, ppm): 5.88–5.86 (m, 1H), 5.12 (m, 2H), 2.78 (t, 2H), 2.54 (q, 2H). ^13^C-NMR (126 MHz, CDCl_3_, δ, ppm): 168.8, 135.8, 116.0, 31.7, 27.9. ^19^F-NMR (300 MHz, CDCl_3_, δ, ppm): −152.91 (2F), −157.53 (2F), −162.10 (1F).

#### 2.3.2. (S)-Perillic Acid Pentafluorophenyl Ester (PerPFP)

PerPFP was synthesized using the same procedure as for PentPFP (see above) using (S)-perillic acid (2 g, 12 mmol). The procedure yielded the product as a colorless oil (3.31 g, 83%).

^1^H-NMR (500 MHz, CDCl_3_, δ, ppm): 7.37 (s, 1H), 4.81–4.76 (d, 2H), 2.61–2.26 (m, 5H), 2.00–1.95 (m, 1H), 1.78 (s, 3H), 1.57 (m, 1H). ^13^C-NMR (126 MHz, CDCl_3_, δ, ppm): 148.2, 144.7, 127.5, 109.6, 39.7, 31.5, 26.7, 24.5, 20.6. ^19^F-NMR (300 MHz, CDCl_3_, δ, ppm): −152.91 (2F), −157.53 (2F), −162.10 (1F).

### 2.4. Polymer Synthesis

PhMI and the active esters were dissolved in either 1,2-dichloroethane (DCE) or 1,1,1,3,3,3-hexafluoro-2-phenyl-2-propanol (HFPP) with AIBN as initiator and *N,N*-dimethylformamide (DMF) or *N,N*-diethylformamide (DEF) as an internal standard. Varying monomer feed ratios (ranging from 1:7 to 7:1, see Tables S1–S4 for details) were used to investigate the kinetics of the copolymerizations and to determine the reactivity ratios. Each solution was degassed by three freeze-pump-thaw cycles, after which the flasks was refilled with argon. A sample was taken for the determination of the initial monomer ratio before heating the reaction mixture to 60 °C.

The conversion of the monomers was monitored by ^1^H-NMR spectroscopy. At regular intervals aliquots were taken and diluted with CDCl_3_ for the NMR measurement. DMF (for copolymerizations in DCE) or DEF (for copolymerizations in HFPP) were used as an internal standard. The decrease of signal intensity of the vinyl groups in PhMI and the active ester in relation to the internal standard was interpreted as the consumption of monomers.

For the series of copolymerization in DCE with a monomer feed of active ester/PhMI 1:2, the polymers formed at higher conversions were purified by precipitation in cold *n*-hexane and subsequent drying *in vacuo*.

### 2.5. Post-Polymerization Functionalization

The obtained polymers were dissolved either in DMF, chloroform or 1,4-dioxane under argon. The appropriate amine was added in excesses ranging from ~1.1 eq. to ~20 eq. (see Tables S7 and S8) compared to the amount of active esters in the copolymers and the resulting mixture was stirred for varying periods of time at elevated temperatures. Functionalized polymers were isolated by precipitation in *n*-hexane, dissolution in chloroform and washing of this organic phase with saturated aq. K_2_CO_3_ solution to remove residual free pentafluorophenol and three rounds of precipitation in *n*-hexane and drying *in vacuo*.

## 3. Results and Discussion

**Figure 1:**
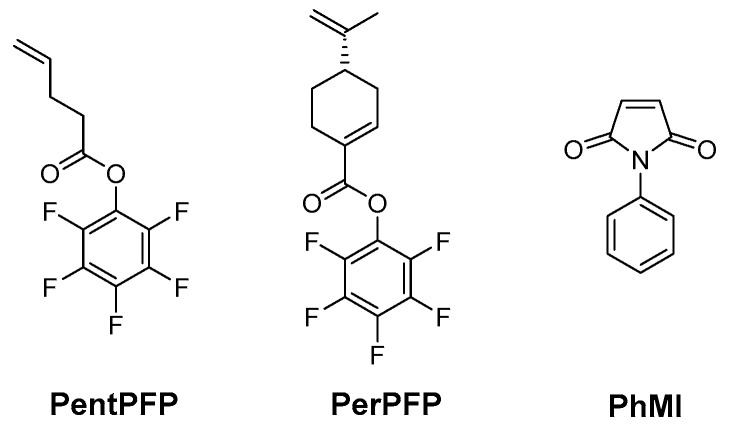
Structures of the monomers used in this work.

Aiming to devise a copolymerization where (a) periodic ABA copolymers are formed and (b) a post-polymerization functionalization of one of the monomers is possible, two different monomers with non-conjugated vinyl groups were designed and prepared. The pentafluorophenyl esters of 4-pentenoic acid (PentPFP) and perillic acid (PerPFP) were chosen. These monomers contain active esters that can easily be functionalized after polymerization using e.g., amines [26,29]. Since the vinyl groups are isolated, it is highly unlikely that these monomers will be able to homopolymerize. Similar to other monomers with non-conjugated vinyl groups [19,20,21,22], these monomers are expected to copolymerize with maleimides though, and for some of these other monomers, periodic ABA copolymers have been found under appropriate conditions, so that similar effects might be expected using PentPFP and PerPFP. *N*-Phenyl maleimide (PhMI) was chosen as the comonomer, primarily since its polymerization rates are relatively high in similar copolymerizations [20]. The structures of the monomers are shown in Figure 1. The copolymerization of these monomers was studied in 1,2-dichloroethane (DCE) and 1,1,1,3,3,3-hexafluoro-2-phenyl-2-propanol (HFPP).

### 3.1. Copolymerization in DCE

Reactivity ratios for the copolymerizations PentPFP and PerPFP with PhMI were determined to investigate the monomer sequence that is formed in the respective copolymers. Copolymerizations were initially performed in DCE, a solvent in which strong penultimate effects, leading to a pronounced tendency for the formation of periodic ABA copolymers, were found for similar monomers [20,21]. The free-radical copolymerizations were initiated by the thermal degradation of AIBN at 60 °C. A series of copolymerizations were performed using feed ratios ranging from 7:1 to 1:7 of the two monomers. Small amounts of DMF were used as an internal standard to be able to monitor the consumption of the monomers. Aliquots were drawn before the start of the polymerization and at regular intervals. The composition of the mixture was investigated by ^1^H-NMR spectroscopy. To be able to accurately determine the consumption of monomers and simultaneously avoid too much compositional drift, conversions of ~20% were targeted for the determination of the reactivity ratios. Under the assumption that all consumed monomer is converted to polymer, the composition of the polymer can be determined for different feed ratios. This data was initially fitted using the terminal model, in which it is assumed that only the last monomer residue in the growing polymer chain influences the addition of the next monomer. A variety of methods have been developed to determine the reactivity ratios, all with their own advantages and disadvantages. In this study, the linear-least-square (LLS), Joshi–Joshi (J–J) [30], Fineman–Ross (F–R) [31], inverted Fineman–Ross (Inv. F–R, in which the definition of monomer 1 and monomer 2 is inverted compared to the normal F–R method), Kelen–Tüdös (K–T) [32], extended Kelen–Tüdös (ext. K–T) [33] and Tidwell–Mortimer (T–M) [34] methods were used. All of these methods except for the latter two assume that no compositional drift takes place, something that is to some degree taken into account in the ext. K–T and T–M methods.

**Figure 2:**
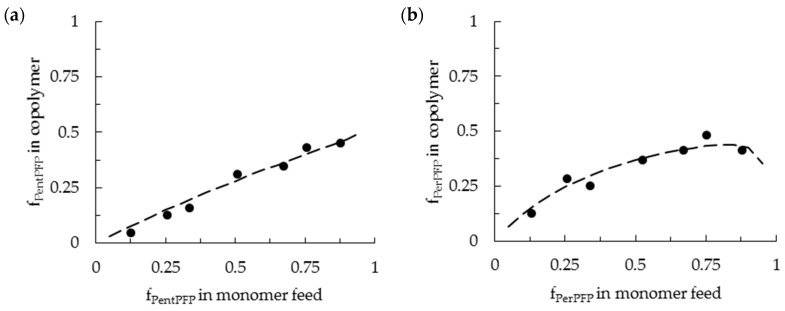
Copolymer composition found at low conversion for a range of different monomer feed ratios ([M_1_]_0_/[PhMI]_0_ = 1/7, 1/3, 1/2, 1/1, 2/1, 3/1, 7/1) for the copolymerization of (**a**) PentPFP/PhMI and (**b**) PerPFP/PhMI with AIBN in DCE at 60 °C. Data points are shown as black dots, the linear-least-square (LLS) fit (dashed lines) is shown as an example.

The polymer compositions found for different feed ratios (and, as an example, the LLS fits) are shown in Figure 2. The reactivity ratios found using the different fitting methods are summarized in Table 1.

As can be seen in Figure 2 (further fits are shown in the Supplementary Materials), the fits describe the polymer compositions fairly well, indicating that the terminal model can satisfactorily describe the copolymerizations of PentPFP with PhMI, and of PerPFP with PhMI in DCE. This also indicates that there are no significant penultimate effects which would lead to the formation of periodic copolymers.

The reactivity ratios (r_1_) of PentPFP and PerPFP (defined as M_1_) are in all cases ~0. Any deviations from 0 are likely within the error margins or correspond to fits that poorly describe the experimental data. This indicates that the homopropagation of these monomers is negligible. For the copolymerization of PentPFP with PhMI r_2_ values around 2 (ranging from 1.6 to 3.5 depending on the method used for the determination of the reactivity ratios) are found. This indicates that the homopropagation of PhMI is slightly favored: if the terminal moiety in the growing chain is a PhMI unit, then there is a slight preference for the addition of further PhMI monomers.

In the copolymerization of PerPFP with PhMI r_2_ values around 0.9 are found, indicating that there is hardly any preference for the addition of PhMI or PerPFP onto a PhMI terminated chain.

**Figure 3:**
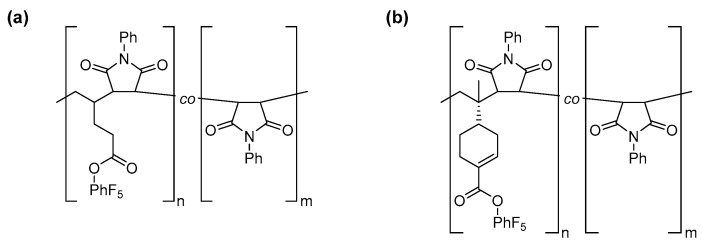
Generalized structures of the copolymers of (**a**) PentPFP or (**b**) PerPFP with PhMI prepared in DCE.

The obtained reactivity ratios indicate that polymers are formed in which the PentPFP or PerPFP residues in a polymer chain are separated by at least one (alternating copolymers would form when large excesses of PentPFP or PerPFP are used in the feed) but often more PhMI units (generalized structures are shown in Figure 3). Interestingly, such copolymers can be used to create polymers in which the average spacing between functional groups can be tuned by changing the feed ratio of the monomers: a more or less linear increase in the PhMI content (and therefore average spacing of active ester moieties along the polymer chain) is observed with increasing fractions of PhMI in the feed.

The difference in the r_2_ values found for the copolymerizations of PentPFP with PhMI and PerPFP with PhMI are likely due to the fact that the vinyl group in PentPFP is less electron-rich than the one in PerPFP (the methyl group in PerPFP exerts a +M effect, increasing electron-density), making PentPFP less reactive in the copolymerization with the e-deficient PhMI.

### 3.2. Copolymerization in HFPP

Since periodic copolymers were not obtained in the copolymerization of either PentPFP or PerPFP with PhMI in DCE, similar copolymerizations were attempted in another solvent, namely HFPP, which has been shown to give rise to even more pronounced penultimate effects in some cases [19,22], perhaps because of a preorganization of monomers due to the binding of the alcohol group to two maleimide monomers.

Therefore, free-radical copolymerizations with different feed ratios of PentPFP or PerPFP with PhMI were also conducted in HFPP. The copolymerizations were performed in a similar manner as in DCE with the difference that *N,N*-diethylformamide (DEF) was used as an internal standard because of its slightly higher boiling point and the fact that the C(=O)H chemical shift is further removed from HFPP signals, resulting in more reliable integration.

The copolymerization behavior of PentPFP with PhMI proceeded in much the same way as in DCE: the composition of the copolymers could satisfactorily be described with the terminal model, which yielded r_1_ and r_2_ values of ~0 and 0.60, respectively (Table 2). This indicates that there are no significant penultimate effects for this monomer in HFPP either, and polymers with similar monomer sequences as shown in Figure 3 are formed. The r_2_ value is much lower in HFPP than in DCE though, indicating that the homopropagation of PhMI is slowed down in HFPP or that the heteropropagation for PentPFP onto a chain-end consisting of a PhMI residue is sped up. This might be a result of hydrogen bonding of the HFPP to the PhMI carbonyl moieties, which reduces the electron density in the vinyl group. The average spacing between active esters will also strongly differ between copolymers prepared at identical feed ratios in DCE and HFPP. The higher r_2_ value in DCE will result in a wider spacing between PentPFP residues as compared to the copolymer prepared in HFPP.

**Figure 4:**
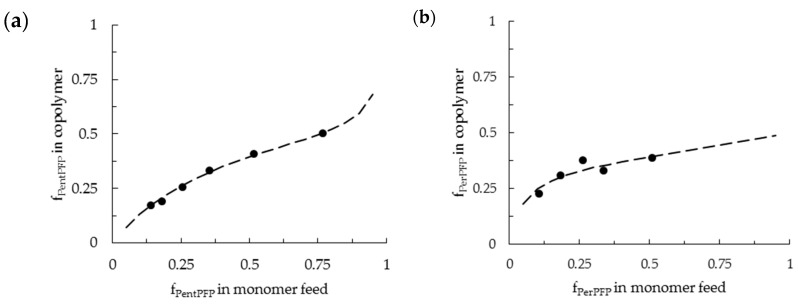
Copolymer composition found at low conversion for a range of monomer feed ratios for the copolymerization of (**a**) PentPFP/PhMI and (**b**) PerPFP/PhMI with AIBN in HFPP at 60 °C. Data points are shown as black dots, the linear-least-square (LLS) fits (dashed line) from the terminal and penultimate model respectively are shown as an example of one of the fits.

One of the first observations while conducting the copolymerizations of PerPFP with PhMI in HFPP was that the rate of polymerization rate was significantly lower than in DCE and also much slower than the copolymerization of PentPFP with PhMI in HFPP. Similar trends were found by Matsuda et al.: for less sterically hindered monomers, faster copolymerization was observed [19]. Extremely slow or negligible copolymerization was found primarily when more PerPFP than PhMI was present in the feed. This resulted in the fact that conversions were not high enough to infer copolymer compositions reliably. Therefore, only feed ratios ranging from 1:1 to 1:7 (PerPFP/PhMI) were used for the determination of reactivity ratios. This data was initially fitted using the terminal model (Table 2), Figure 4a and Supplementary Materials). These fits often resulted in negative reactivity ratios, which are physically impossible, or in very poor fits that did not describe the incorporation of monomers into the polymer satisfactorily. These results indicate that the terminal model describes the copolymerization behavior insufficiently. Therefore, the penultimate model, in which the last two monomer residues in the growing polymer chain are assumed to influence the likelihood of addition of either of the two monomers, was applied. The assumption that the terminal and penultimate monomer residues influence the copolymerization leads to four different possible chain-ends and four reactivity ratios (r_11_, r_21_, r_12_ and r_22_). Since PerPFP cannot significantly homopropagate (as also shown for the copolymerization in DCE), it can be assumed that r_11_ and r_21_ are equal to 0. This simplification makes it possible to determine the remaining reactivity ratios (r_12_ and r_22_) by a modified ext. Kelen–Tüdös procedure or by a linear least square method (Table 2).

**Figure 5:**
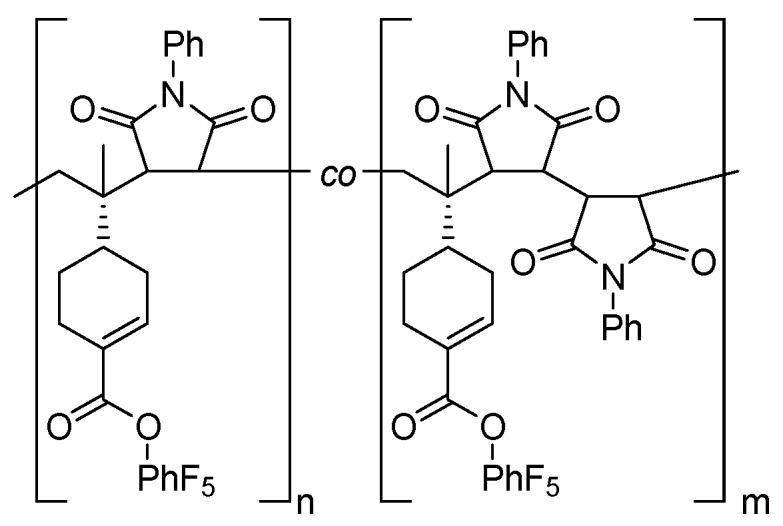
Generalized structures of the copolymer of PerPFP with PhMI prepared in HFPP.

Fitting the experimental data with the penultimate model gave satisfactory fits and physically plausible reactivity ratios (Figure 4). r_12_ values of ~1 were found, indicating that no preference for the addition of either monomer exists when the penultimate and terminal monomer residues were PerPFP and PhMI respectively. The low r_22_ values (~0.1) indicate that the addition of a PerPFP monomer onto a chain-end with two PhMI residues is strongly preferred over the addition of another PhMI monomer, thereby limiting the number of adjacent PhMI moieties to two in most cases, so that a strong tendency towards the formation of periodic ABA copolymers is witnessed. Alternating sequences are not fully excluded, but since copolymerization with large fractions of PerPFP lead to severely retarded polymerization they are hard to obtain. At higher PhMI fractions, ABA-periodic sequences are primarily formed. A generalized structure of this copolymer is shown in Figure 5.

**Figure 6:**
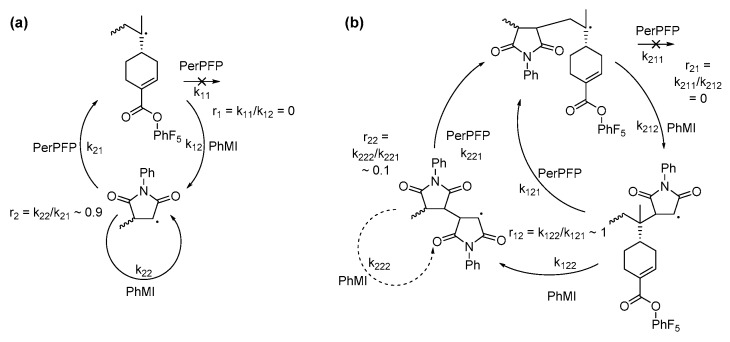
Predominant pathways in the copolymerization of PerPFP with PhMI in (**a**) DCE and (**b**) HFPP.

MALDI-TOF MS and ESI-MS of these copolymers was attempted to further prove the formation of periodic ABA copolymers of PerPFP and PhMI, but desorption/ionization of these polymers was likely very inefficient, resulting in very poor spectra from which no definitive conclusions can be drawn. The predominant reaction pathways found in the copolymerization of PerPFP with PhMI in both DCE and HFPP are summarized in Figure 6.

The fact that strong penultimate effects are found for the copolymerization of PerPFP and PhMI in HFPP, but not for the copolymerization of PentPFP and PhMI, is likely due to the fact that the vinyl group in PerPFP is much more sterically hindered. From literature, it is known that more sterically demanding monomers show more pronounced periodic copolymerization behavior, whereas monomers with less strongly substituted vinyl groups tend to show weaker penultimate effects and mostly tend towards alternating copolymerizations [19]. The vinyl group in PentPFP is much less sterically hindered than the one in PerPFP, and therefore does not show significant penultimate effects (similar as for the copolymerization of 1-alkenes with maleimides), whereas strong penultimate effects are found for PerPFP, which is sterically similar to e.g., limonene [19,22], the copolymerization of which with PhMI has been reported to yield nearly perfect periodic AAB sequences.

### 3.3. Post-Polymerization Functionalization

**Figure 7:**
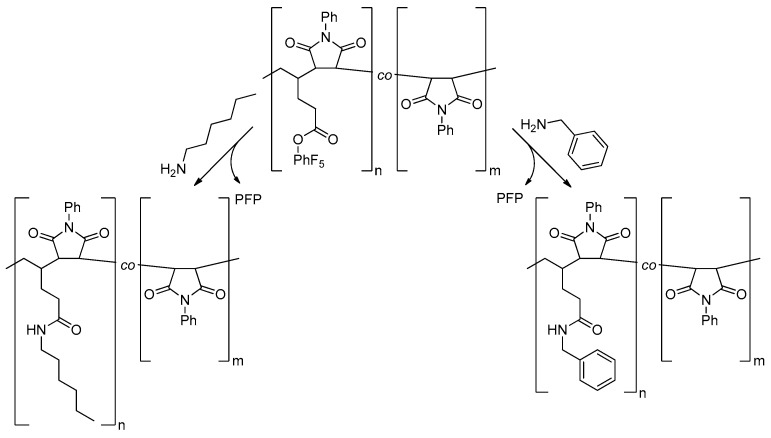
Schematic representation of the post-polymerization functionalization of P(PentPFP-*co*-PhMI) with *n*-hexylamine and benzylamine.

Post-polymerization functionalization experiments were performed for purified P(PentPFP-*co*-PhMI) and P(PerPFP-*co*-PhMI) copolymers prepared in DCE to show that the active esters in the polymers can be functionalized after polymerization (the functionalization reactions for P(PentPFP-*co*-PhMI are schematically shown in Figure 7).

For this proof-of-principle, the copolymers were functionalized with either *n*-hexylamine or benzylamine in DMF, 1,4-dioxane or chloroform. Interestingly, solvatochromic effects were observed during the functionalization of the copolymers: in DMF a very strong purple color was observed, and upon addition of amines the color changed into a deep red. This behavior was not observed in the other solvents. These effects might be caused by interactions between the DMF or the amines with the copolymers [35]. Though these effects were not investigated in detail, similar effects were described by Lam et al. for alternating copolymers of maleic anhydride and vinyl acetate [36].

**Figure 8:**
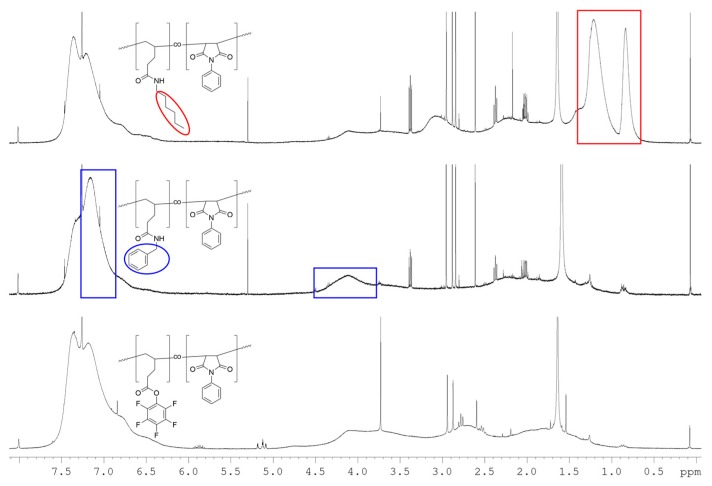
^1^H-NMR spectra of (bottom) P(PentPFP-*co*-PhMI), and after functionalization with (middle) benzylamine or (top) *n*-hexylamine.

A variety of different conditions was attempted, and in all cases the successful and quantitative post-polymerization functionalization of P(PentPFP-*co*-PhMI) and P(PerPFP-*co*-PhMI) could be confirmed by the quantitative disappearance of the characteristic signal pattern in the ^19^F-NMR spectra, even under very mild conditions such as using 1.1 eq. of amine per active ester group at 50 °C. Additionally, major changes in the ^1^H-NMR spectra (Figure 8) of the polymers were observed, corresponding to the introduction of the hexyl (in particular the methyl peak at 0.7–0.95 ppm and the methylene peak between 0.95–1.5 ppm) or benzyl groups (methylene peak at 3.8–4.5 and aromatic signals at 6.9–7.3 ppm) in the polymer. Quantification of the degree of functionalization based on the ^1^H-NMR spectra was not possible since the polymer peaks (especially the backbone signals) are extremely broad and exact integration of signals is difficult. SEC data confirmed that the (apparent) molecular weight of the polymers decreased (shift to higher elution volumes, see Supplementary Materials), as is to be expected since the PFP moiety has a higher molecular weight than either *n*-hexylamine or benzylamine. This shift is more pronounced for benzylamine, which is likely due to solvent effects.

## 4. Conclusions

In conclusion, the copolymerization of non-conjugated vinyl monomers with an active ester moiety with PhMI were investigated, aiming to prepare periodic ABA copolymers in which one of the monomers can be functionalized after polymerization. In DCE, the solvent polymers were formed where the active esters were separated by at least one PhMI unit. The average number of separating PhMI units could be controlled by varying the feed ratio of the monomers. The copolymerization of a sterically demanding non-conjugated vinyl monomer with PhMI in HFPP changed the copolymerization kinetics towards a periodic copolymerization in which preferentially two PhMI separate the PerPFP moieties. Functional (periodic) copolymers with defined spacing of functional groups were easily accessible through the modular post-polymerization amidation of the active esters with primary amines.

The copolymerizations of PhMI with non-conjugated active ester-containing monomers can be used to prepare a wide range of well-defined functional materials with varying spacing of functional groups along the polymer chain.

## Figures and Tables

**Table 1:** Reactivity ratios for the copolymerization of PentPFP and PerPFP (defined as M_1_) and PhMI (M_2_) in DCE using the terminal model.

PentPFP/PhMI	LLS	J–J	F–R	Inv. F–R	K–T	Ext. K–T	T–M
r_1_	0.00	0.19	0.01	0.32	0.10	−0.02	−0.01
r_2_	1.57	2.52	1.92	2.79	2.32	2.40	3.48
r_1_·r_2_	0.01	0.49	0.02	0.89	0.24	−0.04	−0.021
**PerPFP/PhMI**	**LLS**	**J–J**	**F–R**	**Inv. F–R**	**K–T**	**Ext. K–T**	**T–M**
r_1_	0.00	−0.07	−0.03	0.03	−0.06	0.00 ^a^	−0.01
r_2_	0.85	0.75	1.04	0.83	0.75	0.75 ^a^	0.87
r_1_·r_2_	0.00	−0.05	−0.03	0.2	−0.05	0.00 ^a^	−0.01

^a^ For the ext. K-T model the reactions with ratios of 1/7 and 7/1 were excluded.

**Table 2:** Reactivity ratios for the copolymerization of PentPFP (defined as M_1_) with PhMI (M_2_) in HFPP as determined using the terminal model and for the copolymerization of PerPFP (M_1_) and PhMI (M_2_) in HFPP using the penultimate model.

Terminal model		LLS	J–J	F–R	Inv. F–R	K–T	Ext. K–T	T–M
**PentPFP/PhMI**	r_1_	0.06	0.09	0.06	0.03	0.12	0.00	0.01
	r_2_	0.63	0.66	0.64	0.64	0.64	0.55	0.62
	r_1_∙r_2_	0.04	0.06	0.04	0.02	0.05	0.08	0.01
**Penultimate model**		**LLS**					**Ext. K–T**	
**PerPFP/PhMI**	r_11_	0 ^a^					0 ^a^	
	r_21_	0 ^a^					0 ^a^	
	r_12_	0.92					1.16	
	r_22_	0.14					0.11	

^a^ These values were assumed to be 0.

## Supplementary Materials

The following are available online at www.mdpi.com/2073-4360/9/5/166/s1: Characterization data of monomers, details of the copolymerizations, fits of the copolymerizations, characterization of polymers. Figure S1: ^1^H-NMR (CDCl_3_, 500 MHz) spectrum of PentPFP. Figure S2: ^13^C-NMR (CDCl_3_, 126 MHz) spectrum of PentPFP, Figure S3: ^1^H-NMR (CDCl_3_, 500 MHz) spectrum of PerPFP, Figure S4: ^13^C-NMR (CDCl_3_, 126 MHz) spectrum of PerPFP, Figure S5: Fits obtained for the copolymerizations of PentPFP and PhMI in DCE using the (a) curve fitting, (b) Joshi-Joshi, (c) Fineman-Ross, (d) inverted Fineman-Ross, (e) Kelen-Tüdös, (f) extended Kelen-Tüdös and (g) Tidwell-Mortimer methods, Figure S6: Fits obtained for the copolymerizations of PerPFP and PhMI in DCE using the (a) curve fitting, (b) Joshi-Joshi, (c) Fineman-Ross, (d) inverted Fineman-Ross, (e) Kelen-Tüdös, (f) extended Kelen-Tüdös and (g) Tidwell-Mortimer methods, Figure S7: Fits obtained for the copolymerizations of PentPFP and PhMI in HFPP when applying the terminal model, using the (a) curve fitting, (b) Joshi-Joshi, (c) Fineman-Ross, (d) inverted Fineman-Ross, (e) Kelen-Tüdös, (f) extended Kelen-Tüdös and (g) Tidwell-Mortimer methods, Figure S8: Fits obtained for the copolymerizations of PerPFP and PhMI in HFPP when applying the terminal model, using the (a) curve fitting, (b) Joshi-Joshi, (c) Fineman-Ross, (d) inverted Fineman-Ross, (e) Kelen-Tüdös, (f) extended Kelen-Tüdös and (g) Tidwell-Mortimer methods, Figure S9: Fits obtained for the copolymerizations of PerPFP and PhMI in HFPP when applying the penultimate model, using the (a) curve fitting and (b) Kelen- Tüdös methods respectively, Figure S10: ^1^H-NMR (CDCl_3_, 500 MHz) spectrum of P(PentPFP-*co*-PhMI) prepared in DCE with a feed ratio of 1:1 of PentPFP and PhMI, Figure S11: ^19^F-NMR (CDCl_3_, 282 MHz) spectrum of P(PentPFP-*co*-PhMI) prepared in DCE with a feed ratio of 1:1 of PentPFP and PhMI, Figure S12: ^1^H-NMR (CDCl_3_, 500 MHz) spectrum of P(PerPFP-*co*-PhMI) prepared in DCE with a feed ratio of 1:2 of PerPFP and PhMI, Figure S13: ^13^C-NMR (CDCl_3_, 126 MHz) spectrum of P(PerPFP-*co*-PhMI) prepared in DCE with a feed ratio of 1:2 of PerPFP and PhMI, Figure S14: ^19^F-NMR (CDCl_3_, 282 MHz) spectrum of P(PerPFP-*co*-PhMI) prepared in DCE with a feed ratio of 1:2 of PerPFP and PhMI, Figure S15: Molecular weight distribution as determined by SEC (eluent: THF, PS standards) of P(PentPFP-*co*-PhMI) prepared in DCE with a feed ratio of 1:2 of PentPFP and PhMI, Figure S16: Molecular weight distribution as determined by SEC (eluent: THF, PS standards) of P(PerPFP-*co*-PhMI) prepared in DCE with a feed ratio of 1:2 of PerPFP and PhMI, Figure S17: Molecular weight distribution as determined by SEC (eluent: THF, PS standards) of P(PentPFP-*co*-PhMI) prepared in HFPP with a feed ratio of 1:2 of PentPFP and PhMI, Figure S18: ^1^H-NMR (CDCl_3_, 500 MHz) spectrum of P(PentPFP-*co*-PhMI) functionalized with *n*-hexylamine in DMF, Figure S19: ^1^H-NMR (CDCl_3_, 300 MHz) spectrum of P(PentPFP-*co*-PhMI) functionalized with *n*-hexylamine in 1,4-dioxane using microwave irradiation, Figure S20: ^13^C-NMR (CDCl_3_, 126 MHz) spectrum of P(PentPFP-*co*-PhMI) functionalized with *n*-hexylamine in DMF, Figure S21: ^1^H-NMR (CDCl_3_, 500 MHz) spectrum of P(PentPFP-*co*-PhMI) functionalized with benzylamine in DMF, Figure S22: ^13^C-NMR (CDCl_3_, 126 MHz) spectrum of P(PentPFP-*co*-PhMI) functionalized with benzylamine in DMF, Figure S23. SEC elugram (eluent: THF) of P(PentPFP-*co*-PhMI) prepared in DCE with a feed ratio of 1:2 of PentPFP and PhMI before (black) and after (red) functionalization with *n*-hexylamine, Figure S24. SEC elugram (eluent: THF) of P(PentPFP-*co*-PhMI) prepared in DCE with a feed ratio of 1:2 of PentPFP and PhMI before (black) and after (red) functionalization with benzylamine, Figure S25: SEC elugram (eluent: THF) of P(PerPFP-*co*-PhMI) prepared in DCE with a feed ratio of 1:2 of PerPFP and PhMI before (black) and after (red) functionalization with *n*-hexylamine, Figure S26: SEC elugram (eluent: THF) of P(PerPFP-*co*-PhMI) prepared in DCE with a feed ratio of 1:2 of PerPFP and PhMI before (black) and after (red) functionalization with *n*-benzylamine, Table S1: Details for the free-radical copolymerizations of PentPFP and PhMI in DCE/DMF (92:8), Table S2: Details for the free-radical copolymerizations of PerPFP and PhMI in DCE/DMF (92:8), Table S3: Details for the free-radical copolymerizations of PentPFP and PhMI in HFPP with 0.1 eq. *N,N*-diethylformamide as internal standard, Table S4: Details for the free-radical copolymerizations of PerPFP and PhMI in HFPP with 0.1 eq. *N,N*-diethylformamide as internal standard, Table S5: Reactivity ratios for the copolymerization of PerPFP (defined as M_1_) and PhMI (M_2_) in HFPP using the terminal model, Table S6: Molecular weights and dispersity found by SEC in THF with commercially available PS standards, Table S7: Amounts of polymer and amines for the functionalization of P(PerPFP-*co*-PhMI) and P(PentPFP-*co*-PhMI) in 1.5 mL dry 1,4-dioxane at 50 °C for 15 h, Table S8: Amounts of polymer and amines and reaction conditions for the functionalization of P(PentPFP-*co*-PhMI) in various solvents.
