# Supplementary material for: Synthesis and Functionalization of Periodic Copolymers
Source: Polymers (Basel). 2017 May 6;9(5):166. doi: 10.3390/polym9050166 (PMC6432474; doi:10.3390/polym9050166)
Supplement: Supplementary file 1 [file polymers-09-00166-s001.pdf]

Supplementary Materials: Synthesis and Functionalization of Periodic Copolymers

Falk Kubatzki, Lucas Al-Shok and Niels ten Brummelhuis

1. Characterization of Monomers

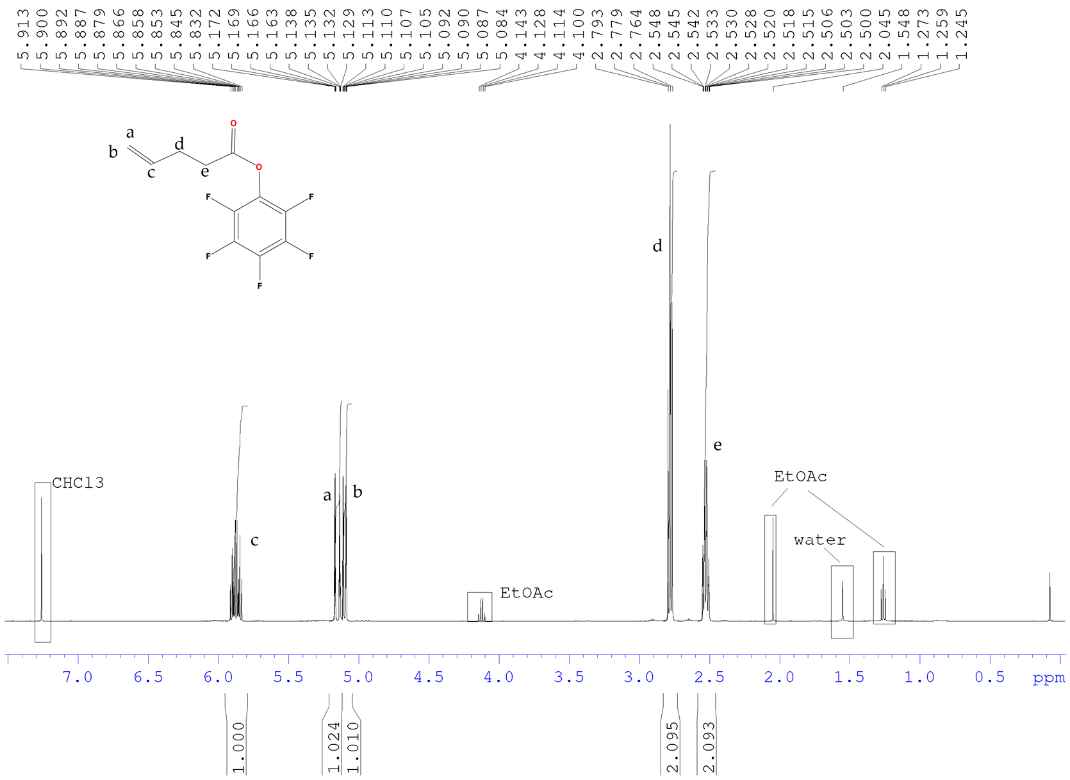

Figure S1. ^{1}H-NMR ($CDCl_3$, 500 MHz) spectrum of PentPFP.

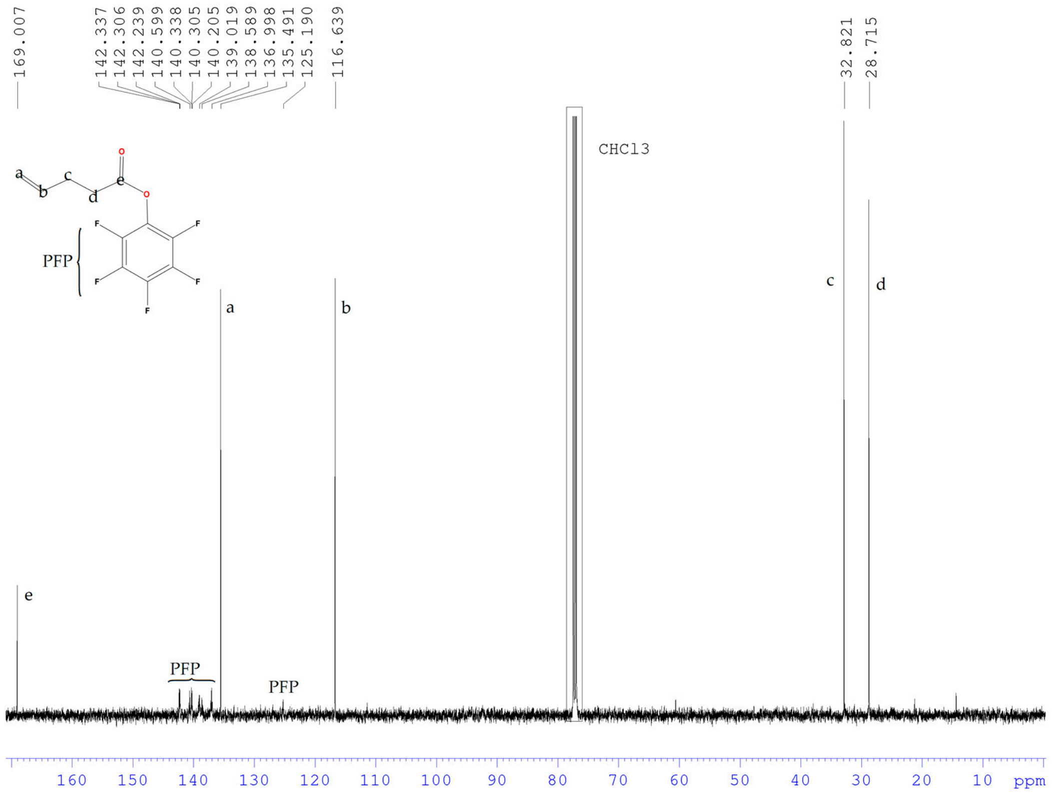

Figure S2. ^{13}C-NMR ($CDCl_3$, 126 MHz) spectrum of PentPFP.

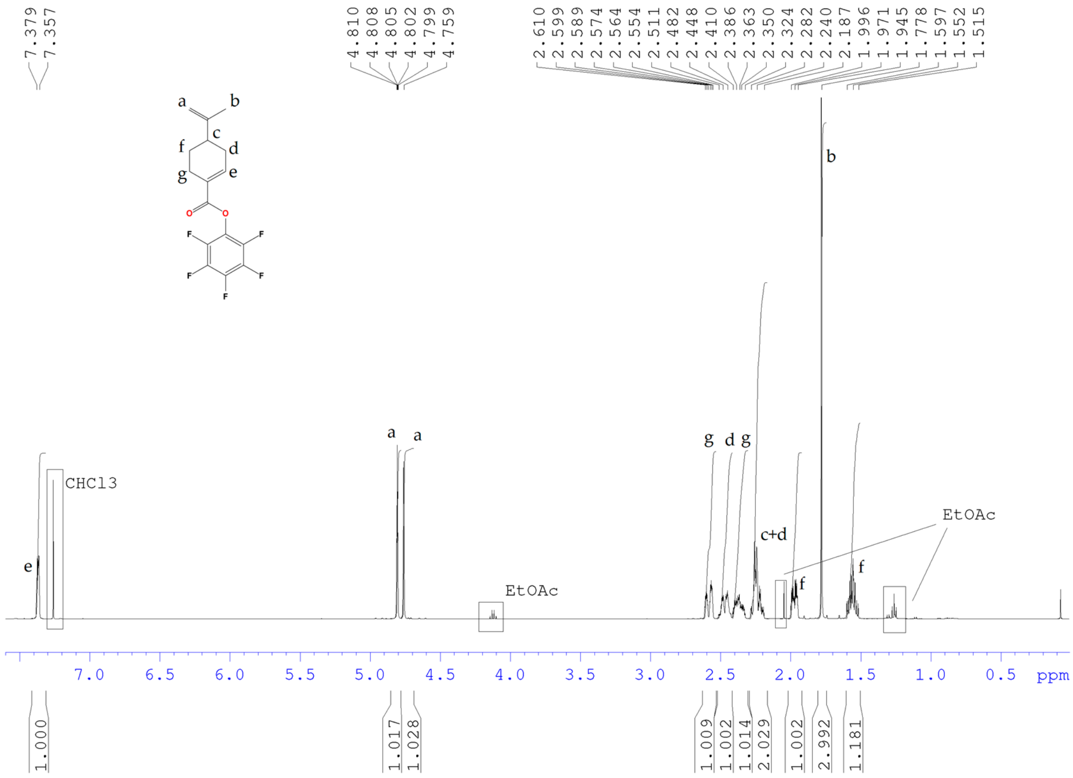

Figure S3. ^{1}H-NMR ($CDCl_3$, 500 MHz) spectrum of PerPFP.

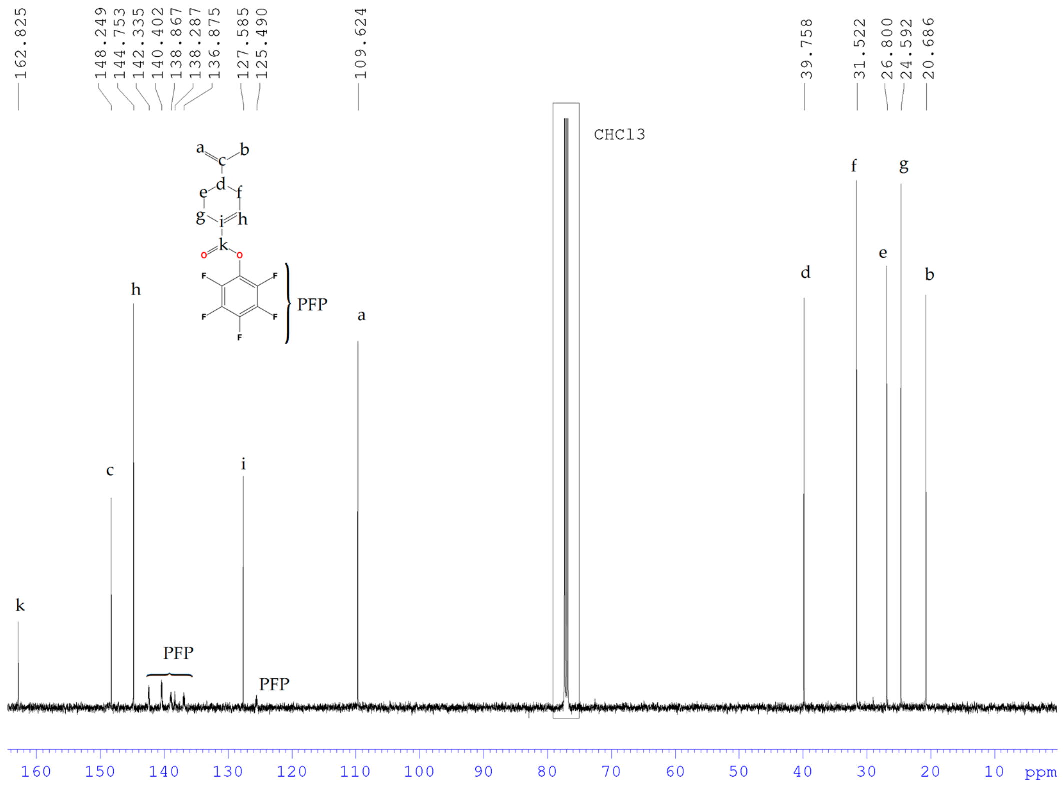

Figure S4. ^{13}C-NMR ($CDCl_3$, 126 MHz) spectrum of PerPFP.

2. Details of Copolymerizations

Table S1. Details for the free-radical copolymerizations of PentPFP and PhMI in DCE/DMF (92:8).

Feed ratio PhMI:PentPFP	PhMI m [mg]	PhMI n [mmol]	PentPFP m [mg]	PentPFP n [mmol]	PhMI+PentPFP n [mmol]	PhMI+PentPFP $eq._{mon}$	AIBN m [mg]	AIBN n [mmol]	AIBN $eq._{initator}$	$V_{reaction}$ [ml]
7:1	343	1.98	78	0.29	2.27	1	17	0.10	0.04	1.5
3:1	295	1.70	149	0.56	2.26	1	30	0.18	0.08	1.5
2:1	261	1.51	199	0.75	2.26	1	15	0.09	0.04	3.0
1:1	409	2.36	628	2.36	4.72	1	30	0.18	0.04	3.1
1:2	263	1.52	801	3.01	4.53	1	30	0.18	0.04	3.0
1:3	100	0.58	452	1.70	2.27	1	30	0.18	0.08	1.5
1:7	49	0.28	524	1.97	2.25	1	30	0.18	0.08	1.5

Table S2. Details for the free-radical copolymerizations of PerPFP and PhMI in DCE/DMF (92:8).

	PhMI		PerPFP		PhMI+PerPFP		AIBN			
Feed ratio PhMI:PerPFP	m [mg]	n [mmol]	m [mg]	n [mmol]	n [mmol]	$eq._{mon}$	m [mg]	n [mmol]	$eq._{initator}$	$V_{reaction}$ [ml]
7:1	342	1.98	94	0.29	2.27	1	30	0.18	0.08	1.5
3:1	292	1.70	186	0.56	2.26	1	30	0.18	0.08	1.5
2:1	520	1.51	503	0.75	2.26	1	30	0.18	0.08	3.0
1:1	391	2.36	748	2.36	4.72	1	15	0.09	0.02	3.1
1:2	260	1.52	1000	3.01	4.53	1	30	0.18	0.04	3.0
1:3	98	0.58	562	1.70	2.27	1	30	0.18	0.08	1.5
1:7	94	0.28	655	1.97	2.25	1	30	0.18	0.08	1.5

Table S3. Details for the free-radical copolymerizations of PentPFP and PhMI in HFPP with 0.1 eq. *N,N*-diethylformamide as internal standard.

	PhMI		PentPFP		PhMI+PentPFP		AIBN			
Feed ratio PhMI:PentPFP	m [mg]	n [mmol]	m [mg]	n [mmol]	n [mmol]	$eq._{mon}$	m [mg]	n [mmol]	$eq._{initator}$	$V_{reaction}$ [ml]
1:3	78	0.45	359	1.35	1.80	1	6	0.036	0.02	1.5
1:1	156	0.90	240	0.90	1.80	1	6	0.036	0.02	1.5
2:1	208	1.20	160	0.60	1.80	1	6	0.036	0.02	1.5
3:1	234	1.35	120	0.45	1.80	1	6	0.036	0.02	1.5
5:1	260	1.50	80	0.30	1.80	1	6	0.036	0.02	1.5
7:1	273	1.58	60	0.23	1.80	1	6	0.036	0.02	1.5

Table S4. Details for the free-radical copolymerizations of PerPFP and PhMI in HFPP with 0.1 eq. *N,N*-diethylformamide as internal standard.

	PhMI		PerPFP		PhMI+PerPFP		AIBN			
Feed ratio PhMI:PerPFP	m [mg]	n [mmol]	m [mg]	n [mmol]	n [mmol]	$eq._{mon}$	m [mg]	n [mmol]	$eq._{initator}$	$V_{reaction}$ [ml]
1:1	156	0.90	299	0.90	1.80	1	6	0.036	0.02	1.5
2:1	208	1.20	199	0.60	1.80	1	6	0.036	0.02	1.5
3:1	234	1.35	150	0.45	1.80	1	6	0.036	0.02	1.5
5:1	260	1.50	100	0.30	1.80	1	6	0.036	0.02	1.5
7:1	273	1.58	75	0.23	1.80	1	6	0.036	0.02	1.5

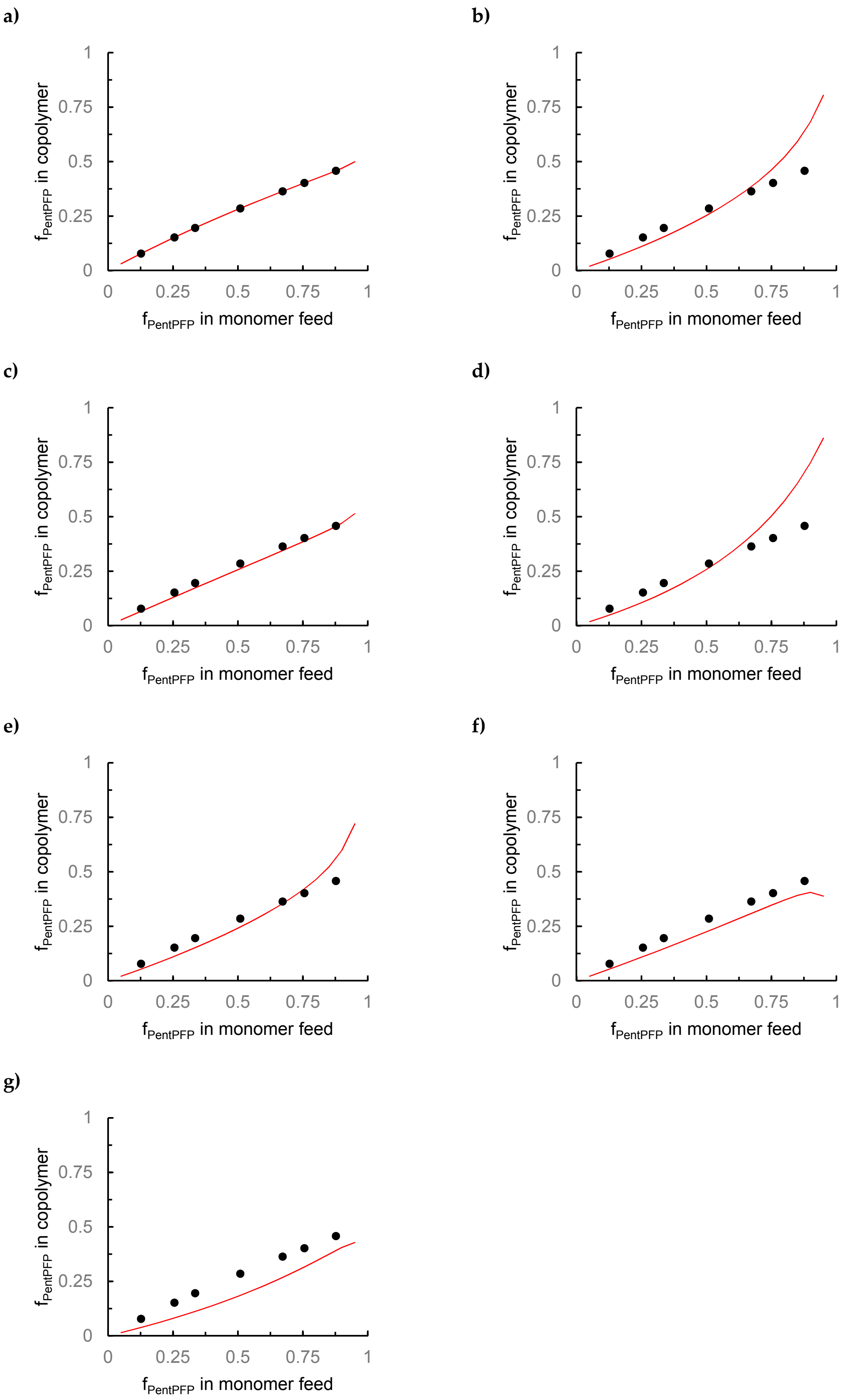

Figure S5. Fits obtained for the copolymerizations of PentPFP and PhMI in DCE using the (**a**) curve fitting, (**b**) Joshi-Joshi, (**c**) Fineman-Ross, (**d**) inverted Fineman-Ross, (**e**) Kelen-Tüdös, (**f**) extended Kelen-Tüdös and (**g**) Tidwell-Mortimer methods.

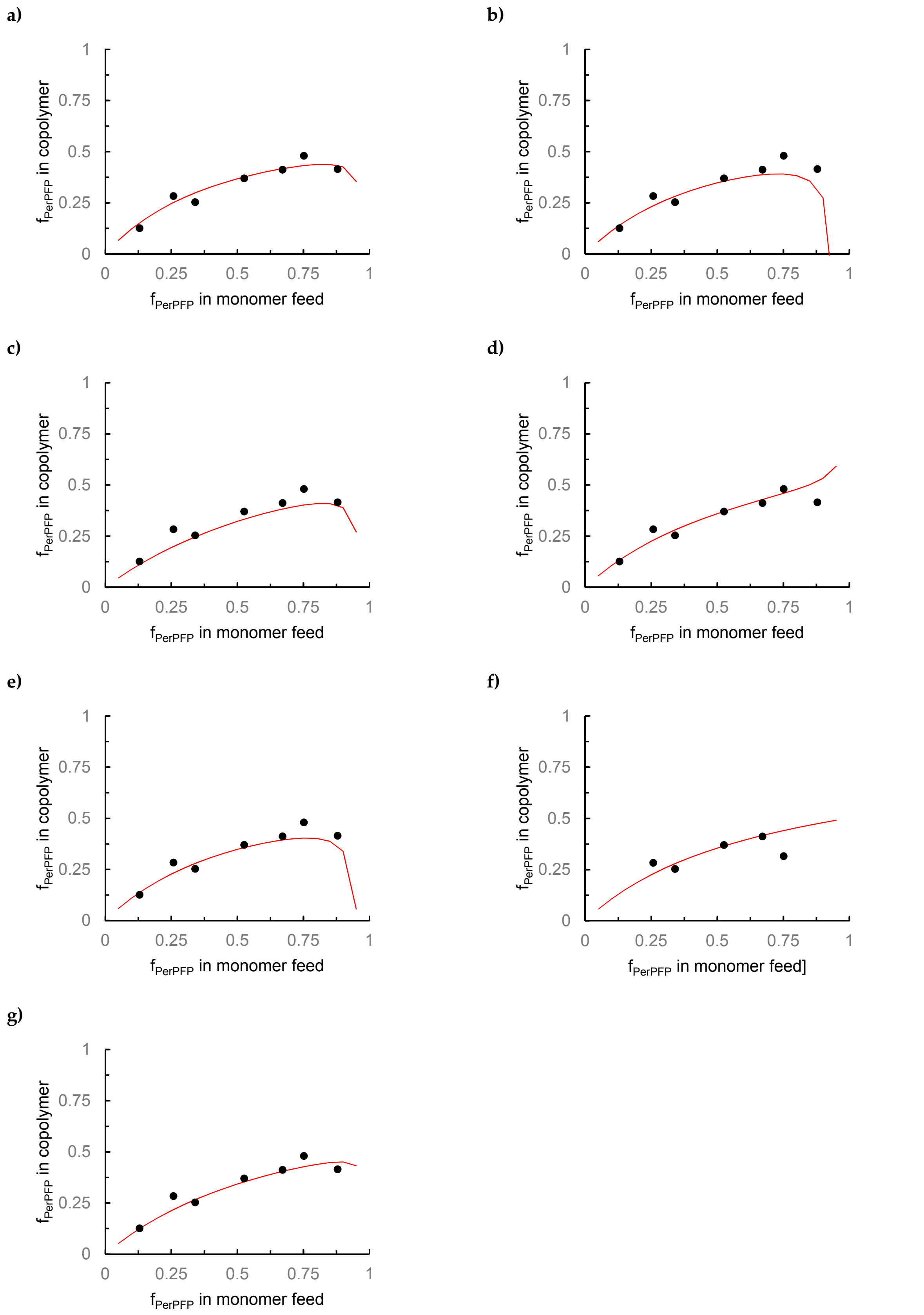

Figure S6. Fits obtained for the copolymerizations of PerPFP and PhMI in DCE using the (**a**) curve fitting, (**b**) Joshi-Joshi, (**c**) Fineman-Ross, (**d**) inverted Fineman-Ross, (**e**) Kelen-Tüdös, (**f**) extended Kelen-Tüdös and (**g**) Tidwell-Mortimer methods.

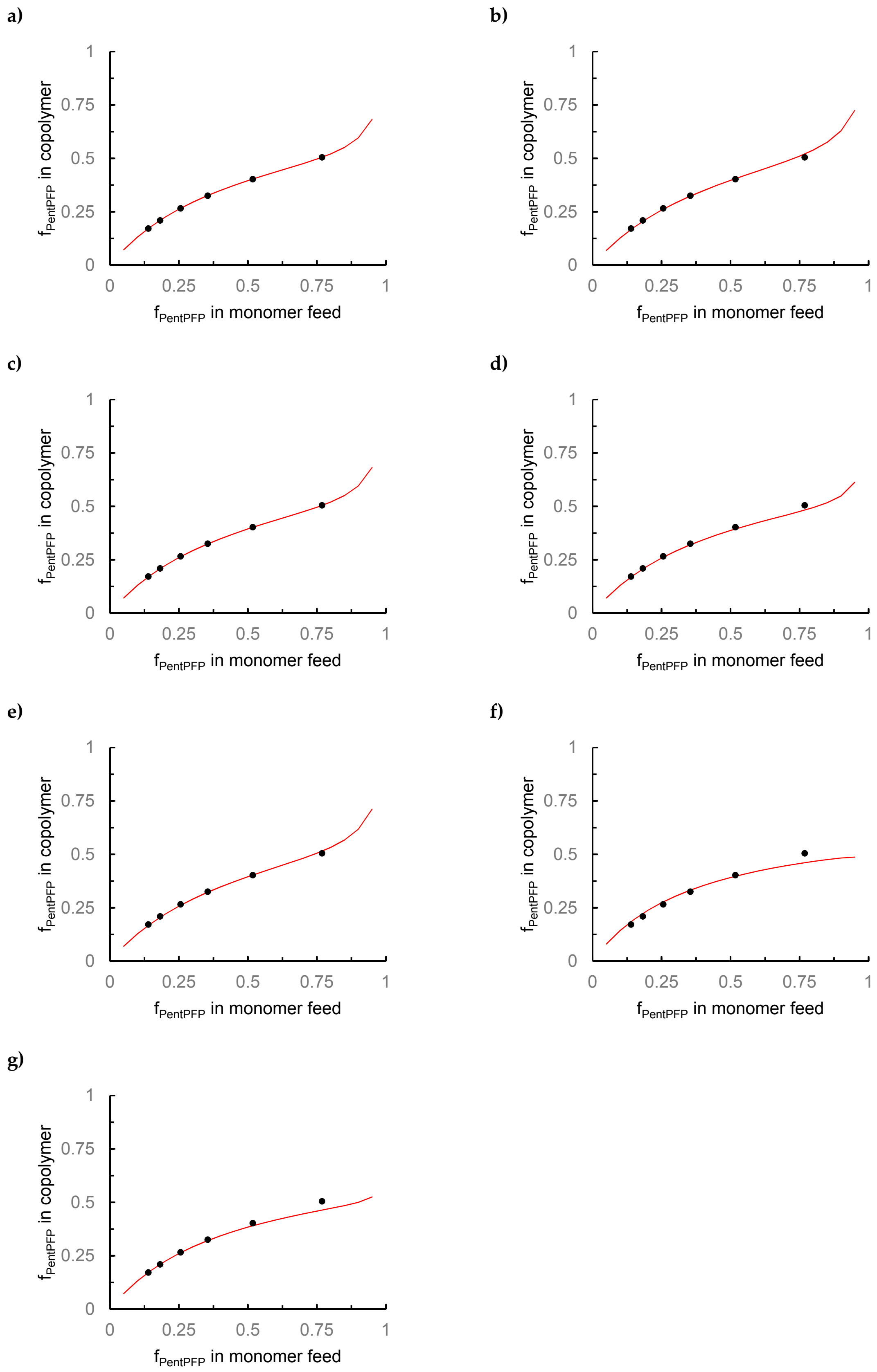

Figure S7. Fits obtained for the copolymerizations of PentPFP and PhMI in HFPP when applying the terminal model, using the (**a**) curve fitting, (**b**) Joshi-Joshi, (**c**) Fineman-Ross, (**d**) inverted Fineman-Ross, (**e**) Kelen-Tüdös, (**f**) extended Kelen-Tüdös and (**g**) Tidwell-Mortimer methods.

Table S5. Reactivity ratios for the copolymerization of PerPFP (defined as M_1) and PhMI (M_2) in HFPP using the terminal model.

Terminal model	LLS	Ext. K-T	J-J	F-R	Inv. F-R	K-T	T-M
r_1	-0.23	0.00	-0.34	-0.21	-0.15	0.11	0.07
r_2	0.26	0.14	0.19	0.272	0.27	0.11	0.10
$r_1 \cdot r_2$	0.00	0.00	-0.06	-0.06	-0.03	0.01	0.01

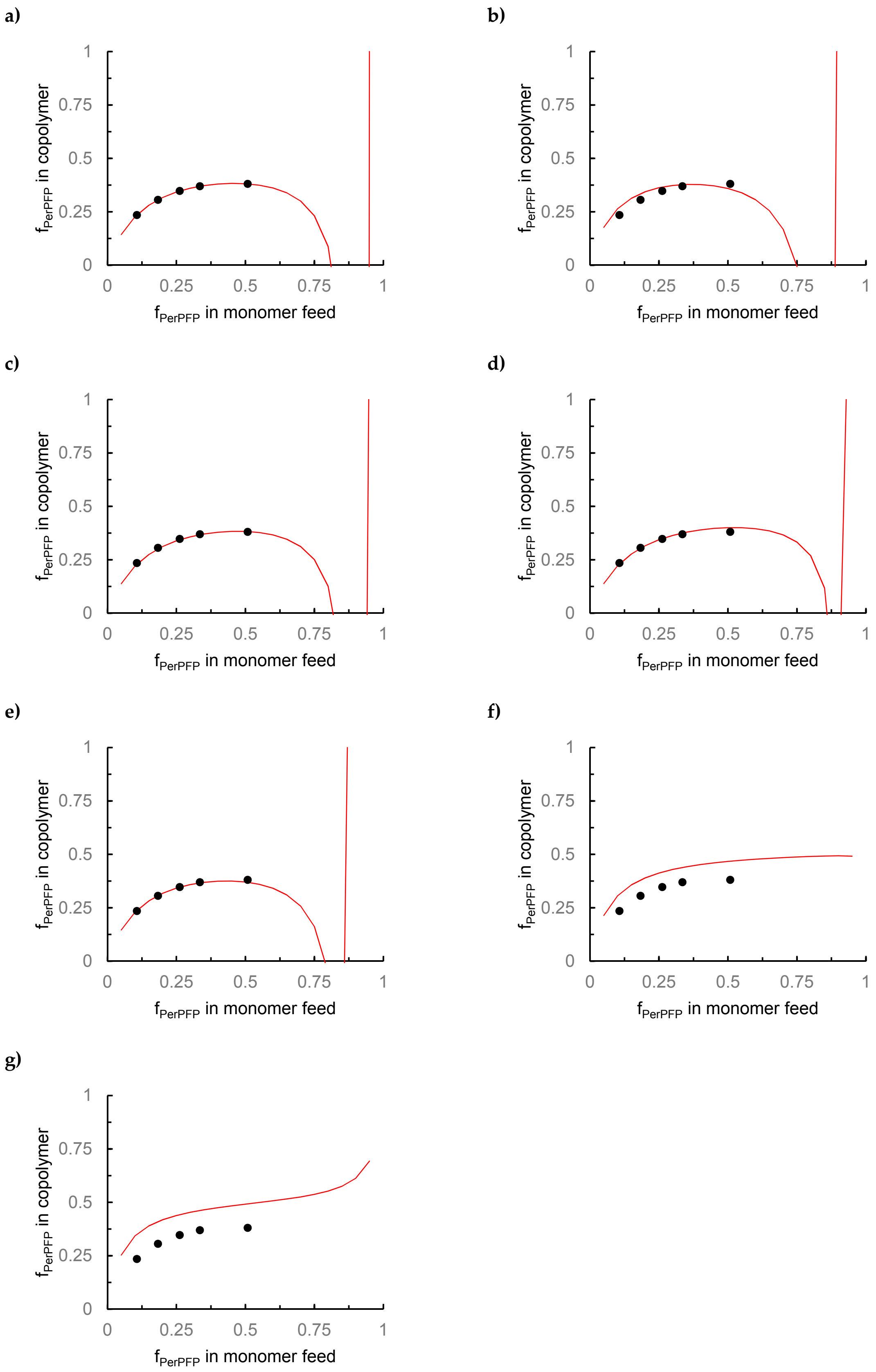

Figure S8. Fits obtained for the copolymerizations of PerPFP and PhMI in HFPP when applying the terminal model, using the (a) curve fitting, (b) Joshi-Joshi, (c) Fineman-Ross, (d) inverted Fineman-Ross, (e) Kelen-Tüdös, (f) extended Kelen-Tüdös and (g) Tidwell-Mortimer methods.

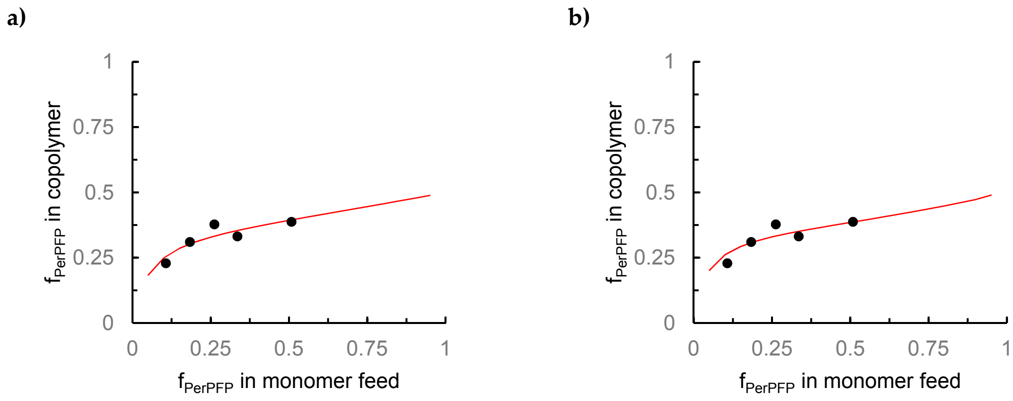

Figure S9. Fits obtained for the copolymerizations of PerPFP and PhMI in HFPP when applying the penultimate model, using the (**a**) curve fitting and (**b**) Kelen-Tüdös methods respectively.

3. Characterization of Polymers

3.1 Before Functionalization

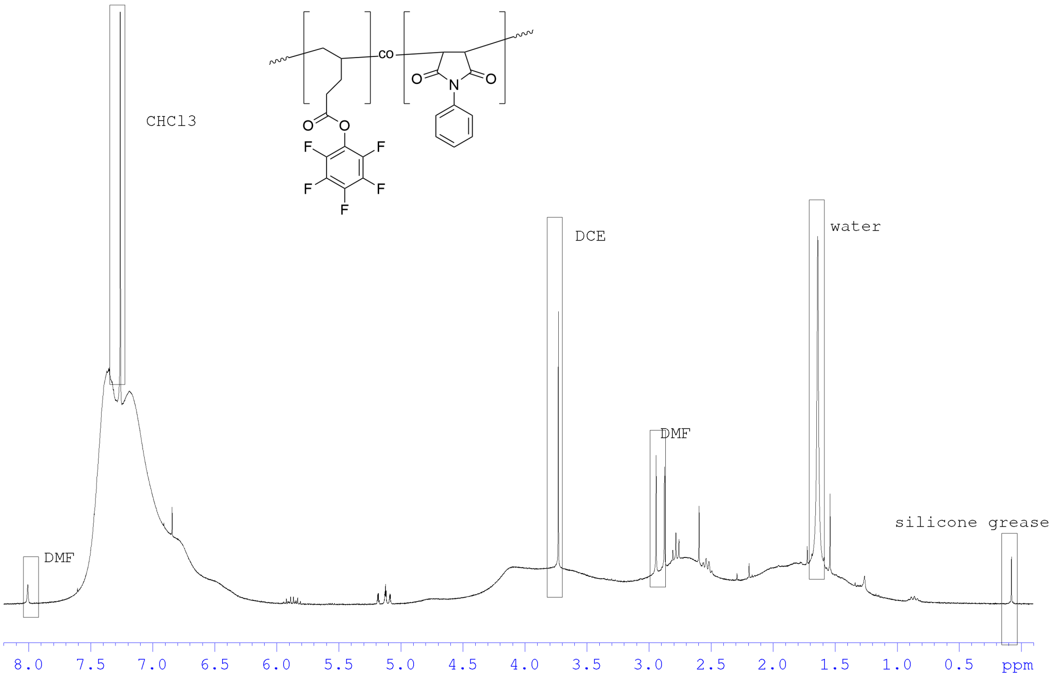

Figure S10. ^{1}H-NMR ($CDCl_3$, 500 MHz) spectrum of P(PentPFP-*co*-PhMI) prepared in DCE with a feed ratio of 1:1 of PentPFP and PhMI.

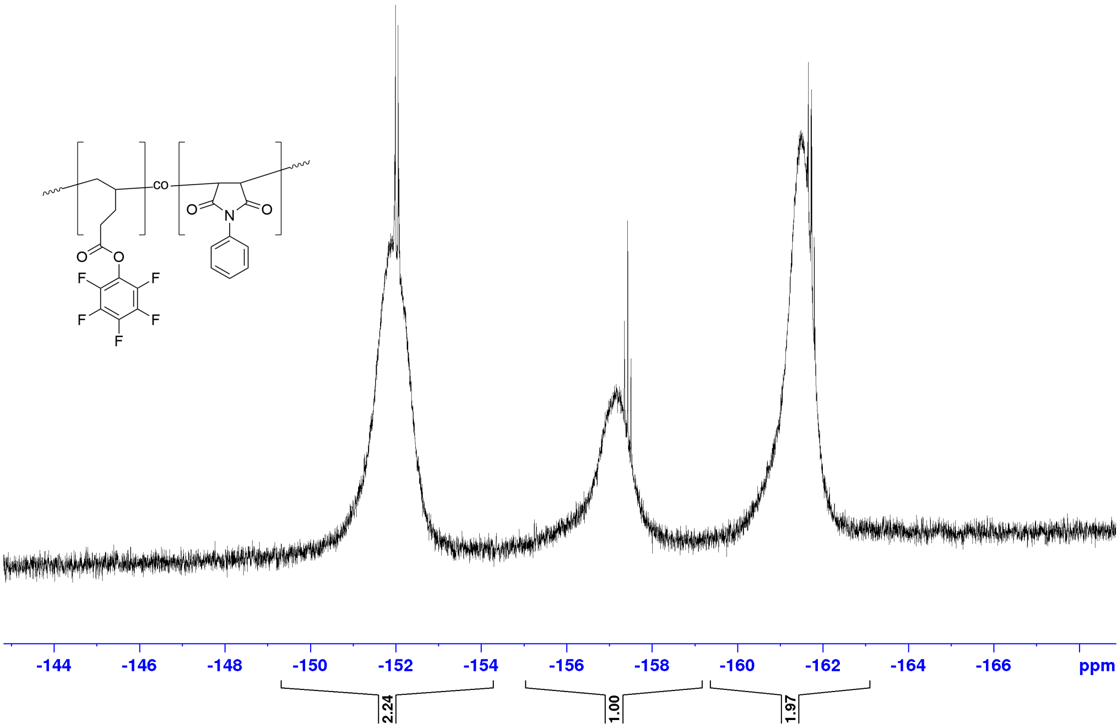

Figure S11. ^{19}F-NMR ($CDCl_3$, 282 MHz) spectrum of P(PentPFP-*co*-PhMI) prepared in DCE with a feed ratio of 1:1 of PentPFP and PhMI.

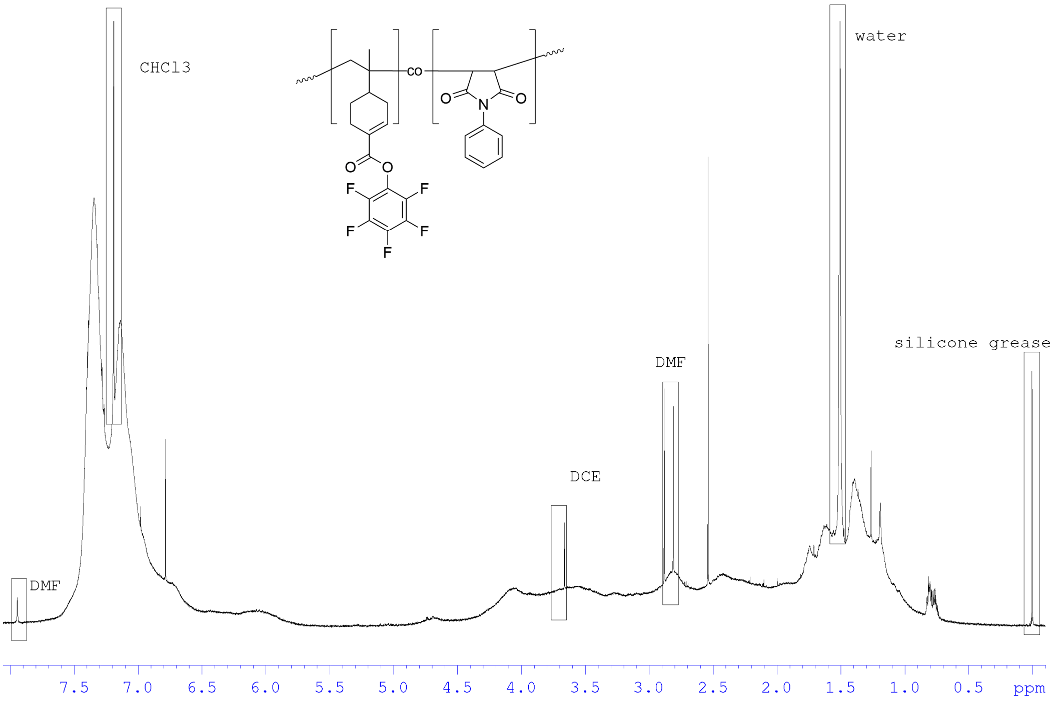

Figure S12. ^{1}H-NMR ($CDCl_3$, 500 MHz) spectrum of P(PerPFP-*co*-PhMI) prepared in DCE with a feed ratio of 1:2 of PerPFP and PhMI.

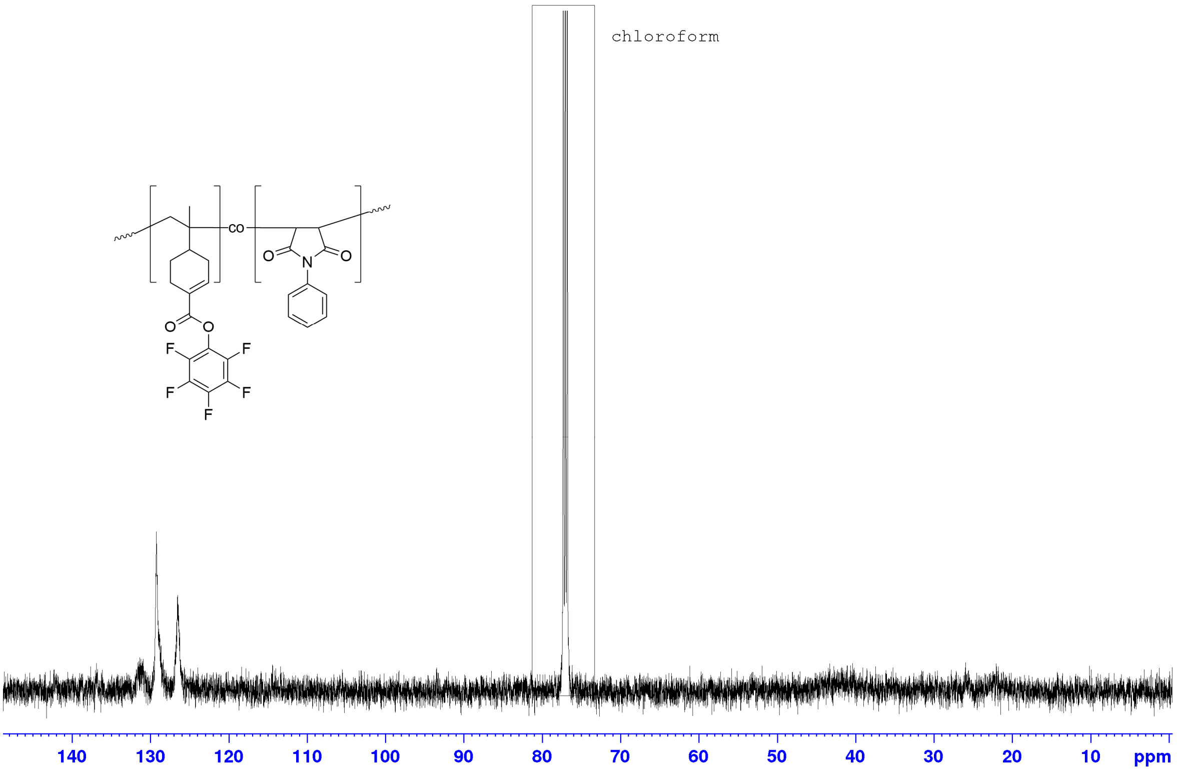

Figure S13. ^{13}C-NMR ($CDCl_3$, 126 MHz) spectrum of P(PerPFP-*co*-PhMI) prepared in DCE with a feed ratio of 1:2 of PerPFP and PhMI.

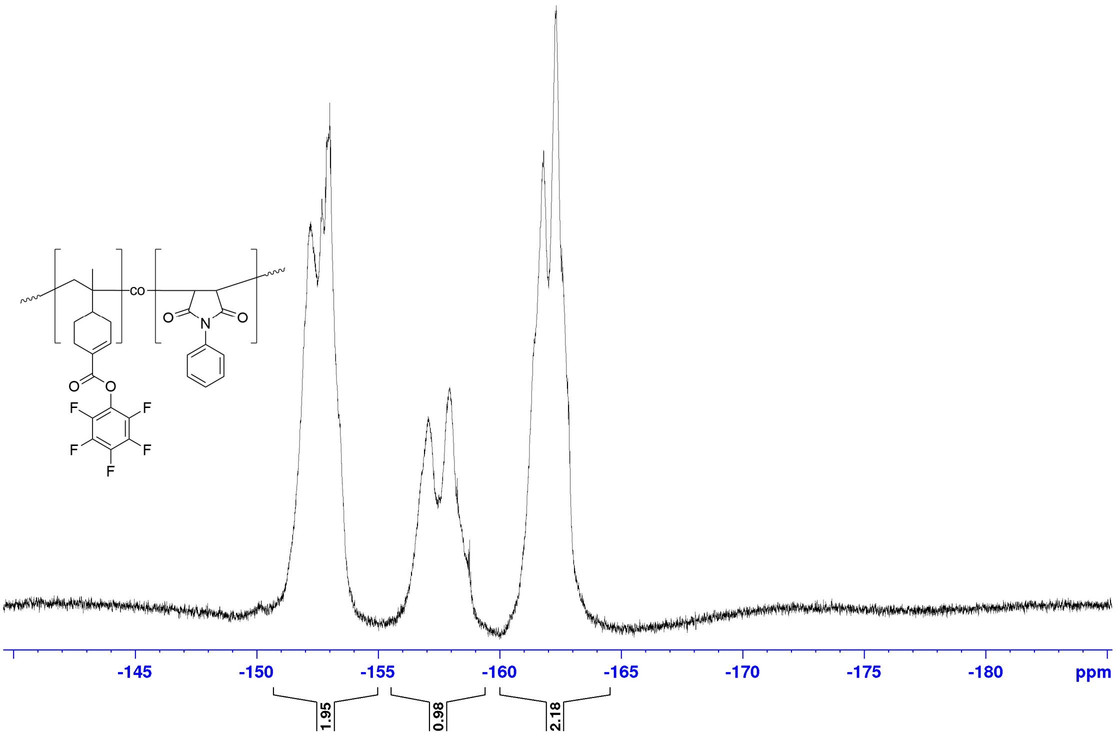

Figure S14. ^{19}F-NMR ($CDCl_3$, 282 MHz) spectrum of P(PerPFP-*co*-PhMI) prepared in DCE with a feed ratio of 1:2 of PerPFP and PhMI.

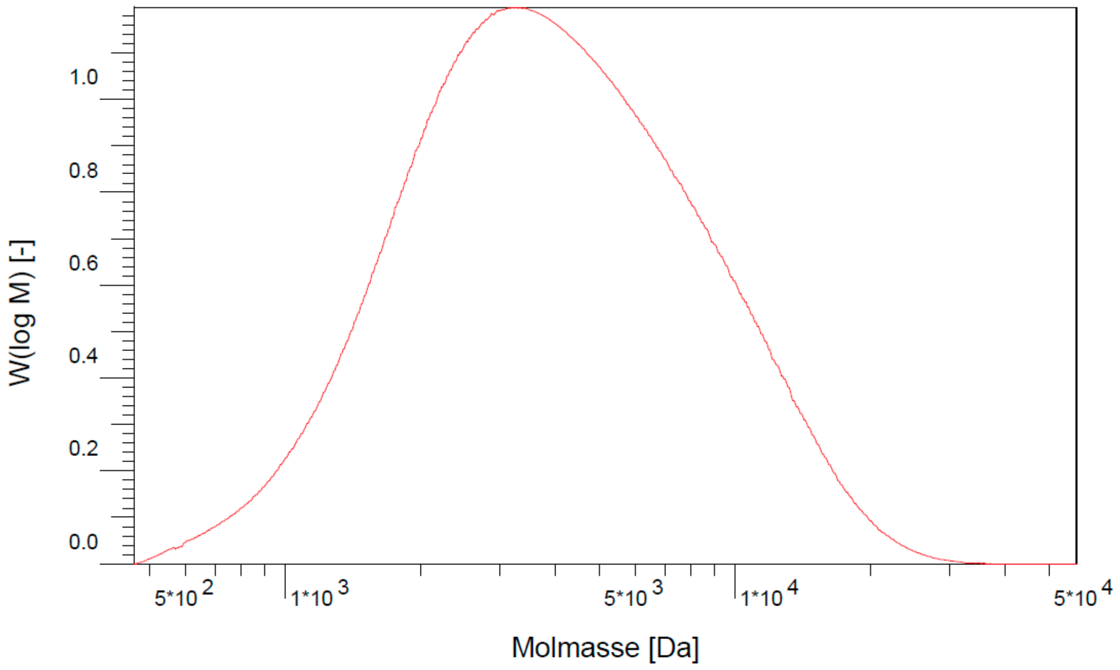

Figure S15. Molecular weight distribution as determined by SEC (eluent: THF, PS standards) of P(PentPFP-*co*-PhMI) prepared in DCE with a feed ratio of 1:2 of PentPFP and PhMI.

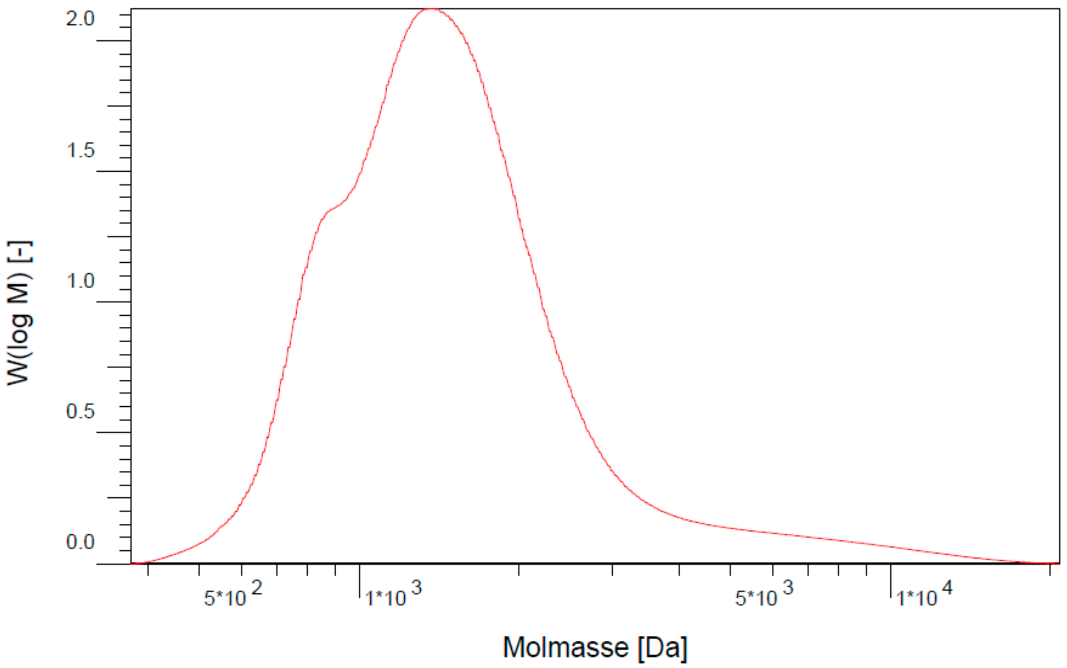

Figure S16. Molecular weight distribution as determined by SEC (eluent: THF, PS standards) of P(PerPFP-*co*-PhMI) prepared in DCE with a feed ratio of 1:2 of PerPFP and PhMI.

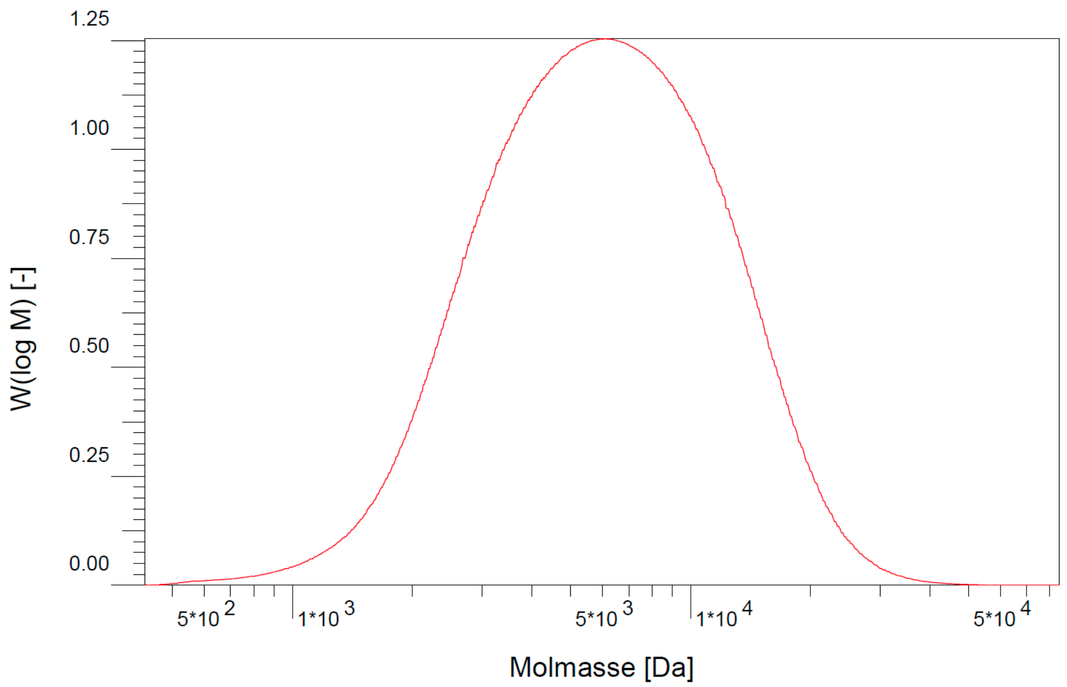

Figure S17. Molecular weight distribution as determined by SEC (eluent: THF, PS standards) of P(PentPFP-*co*-PhMI) prepared in HFPP with a feed ratio of 1:2 of PentPFP and PhMI.

Table S6. Molecular weights and dispersity found by SEC in THF with commercially available PS standards.

Copolymer	Prepared in	M_w [·10³ g/mol]	Đ [-]	M_p [·10³ g/mol]
P(PentPFP-*co*-PhMI)	DCE	5.0	1.67	3.8
P(PerPFP-*co*-PhMI)	DCE	1.8	1.36	1.3
P(PentPFP-*co*-PhMI)	HFPP	7.7	1.56	6.6

3.2 After Functionalization

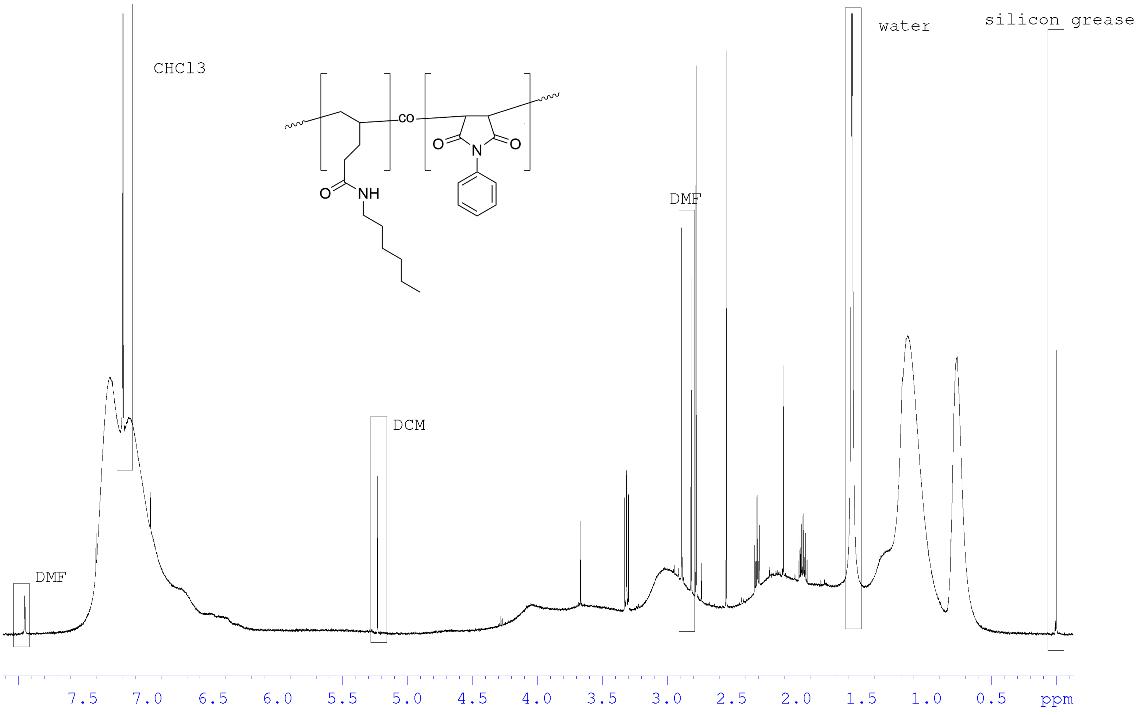

Figure S18. ^{1}H-NMR ($CDCl_3$, 500 MHz) spectrum of P(PentPFP-*co*-PhMI) functionalized with *n*-hexylamine in DMF.

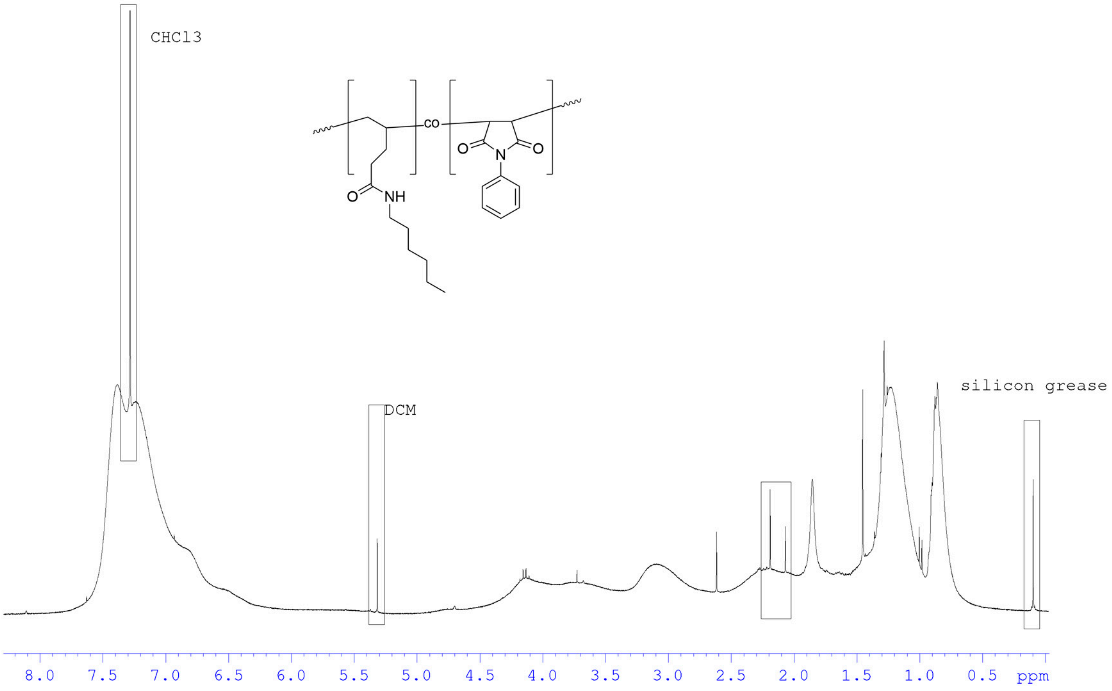

Figure S19. ^{1}H-NMR ($CDCl_3$, 300 MHz) spectrum of P(PentPFP-*co*-PhMI) functionalized with *n*-hexylamine in 1,4-dioxane using microwave irradiation.

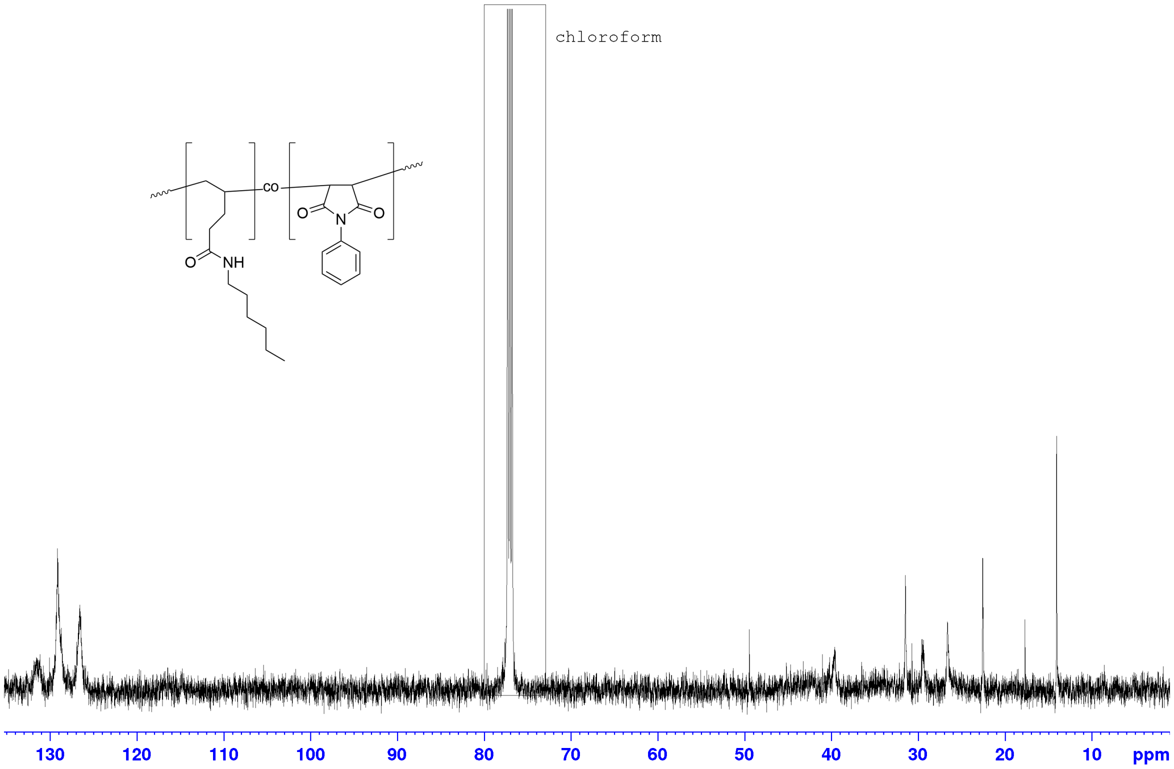

Figure S20. ^{13}C-NMR ($CDCl_3$, 126 MHz) spectrum of P(PentPFP-*co*-PhMI) functionalized with *n*-hexylamine in DMF.

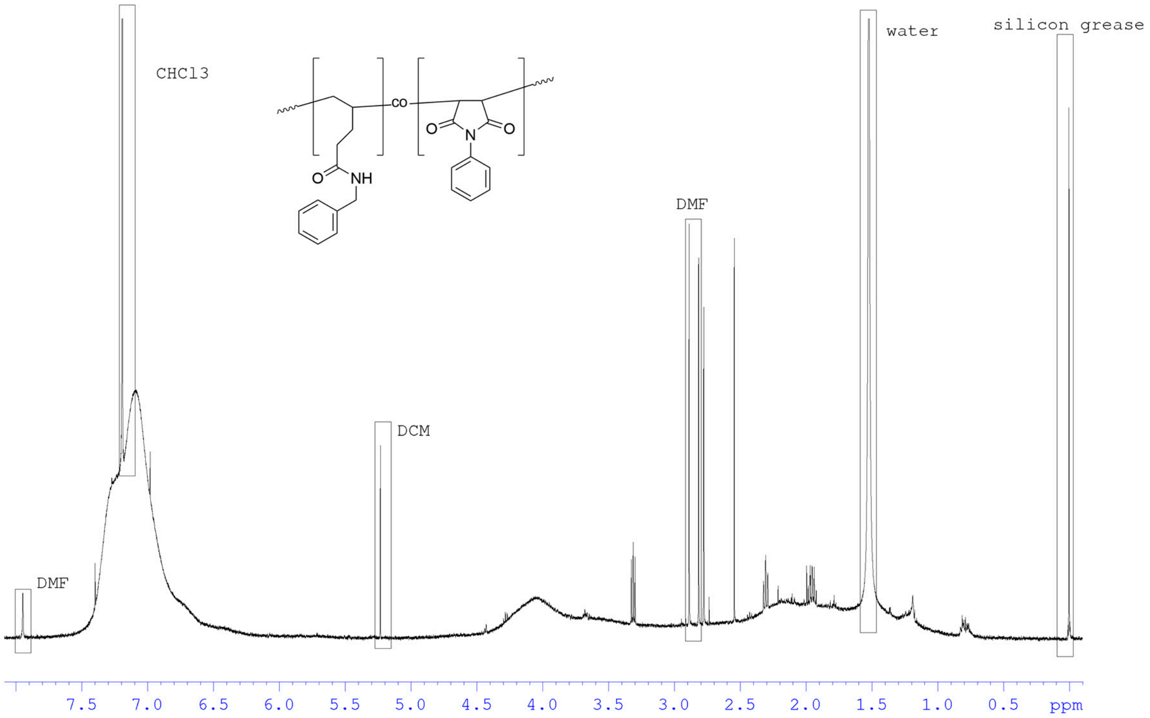

Figure S21. ^{1}H-NMR ($CDCl_3$, 500 MHz) spectrum of P(PentPFP-*co*-PhMI) functionalized with benzylamine in DMF.

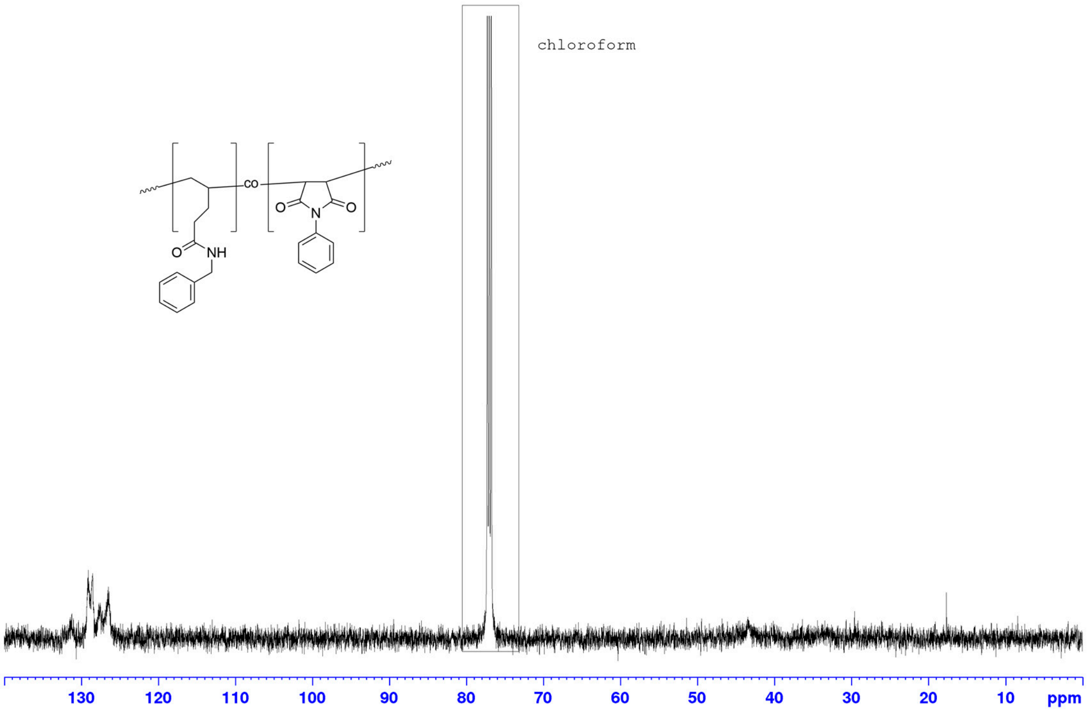

Figure S22. ^{13}C-NMR ($CDCl_3$, 126 MHz) spectrum of P(PentPFP-*co*-PhMI) functionalized with benzylamine in DMF.

Table S7. Amounts of polymer and amines for the functionalization of P(PerPFP-*co*-PhMI) and P(PentPFP-*co*-PhMI) in 1.5 ml dry 1,4-dioxane at 50 °C for 15 h.

Polymer	$m_{polym.}$ [mg]	$n_{PFP\ ester}$ [mmol]	Amine	m_{amine} [mg]	n_{amine} [mmol]	eq.
P(PerPFP-*co*-PhMI)	8	0.012	Hexylamine	0.023	0.22	19.2
	10	0.015	Benzylamine	0.029	0.28	18.7
P(PentPFP-*co*-PhMI)	12	0.020	Hexylamine	0.023	0.22	11.6
	17	0.028	Benzylamine	0.029	0.28	9.9

Table S8. Amounts of polymer and amines and reaction conditions for the functionalization of P(PentPFP-*co*-PhMI) in various solvents.

$m_{polym.}$ [mg]	$n_{PFP\ ester}$ [mmol]	Amine	m_{amine} [mg]	n_{amine} [mmol]	$eq._{amine}$	Solvent	$V_{solvent}$ [ml]	t [h]	T [°C]
41.9	0.068	Hexylamine	0.023	0.23	3.3	$CHCl_3$	3	24	50
43.6	0.071	Hexylamine	0.023	0.23	3.2	1,4-dioxane	3	24	50
43.8	0.072	Benzylamine	0.029	0.28	3.8	$CHCl_3$	3	3.5	60
46.2	0.075	Benzylamine	0.029	0.28	3.6	1,4-dioxane	3	3.5	60
46.0	0.075	Hexylamine	0.023	0.22	3.0	1,4-dioxane	3	0.33 [a]	60
49.6	0.081	Hexylamine	0.009	0.09	1.1	DMF	5	24	50
49.7	0.081	Benzylamine	0.012	0.11	1.4	DMF	5	24	50

[a] Besides the conventional heating by oil bath, faster conversion were achieved by heating under microwave heating with a power of 50 W.

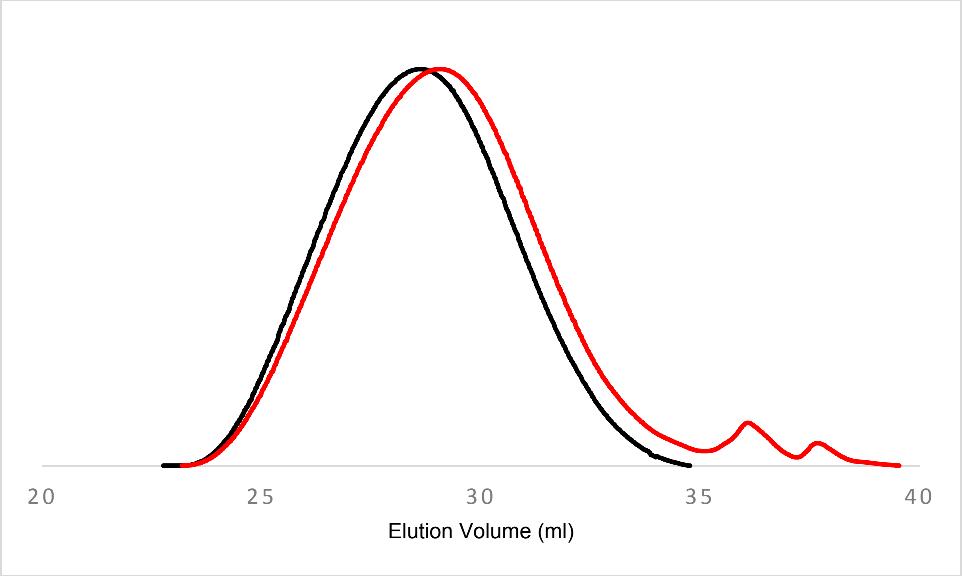

Figure S23. SEC elugram (eluent: THF) of P(PentPFP-*co*-PhMI) prepared in DCE with a feed ratio of 1:2 of PentPFP and PhMI before (black) and after (red) functionalization with *n*-hexylamine.

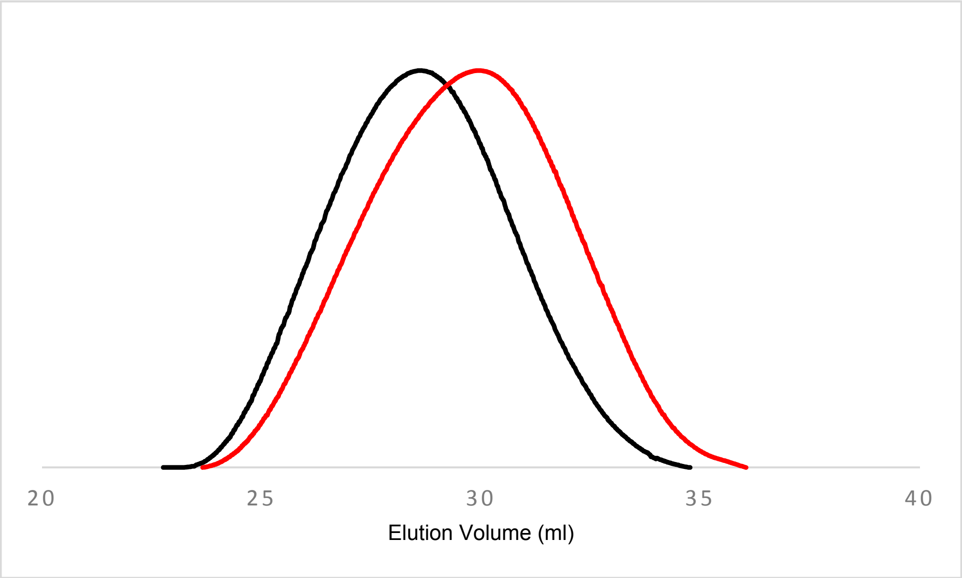

Figure S24. SEC elugram (eluent: THF) of P(PentPFP-*co*-PhMI) prepared in DCE with a feed ratio of 1:2 of PentPFP and PhMI before (black) and after (red) functionalization with benzylamine.

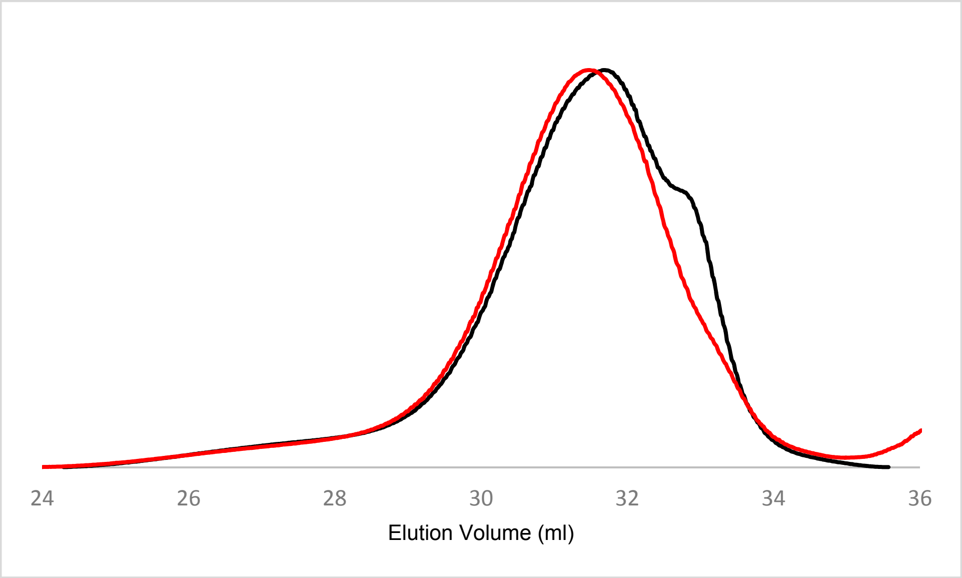

Figure S25. SEC elugram (eluent: THF) of P(PerPFP-*co*-PhMI) prepared in DCE with a feed ratio of 1:2 of PerPFP and PhMI before (black) and after (red) functionalization with *n*-hexylamine.

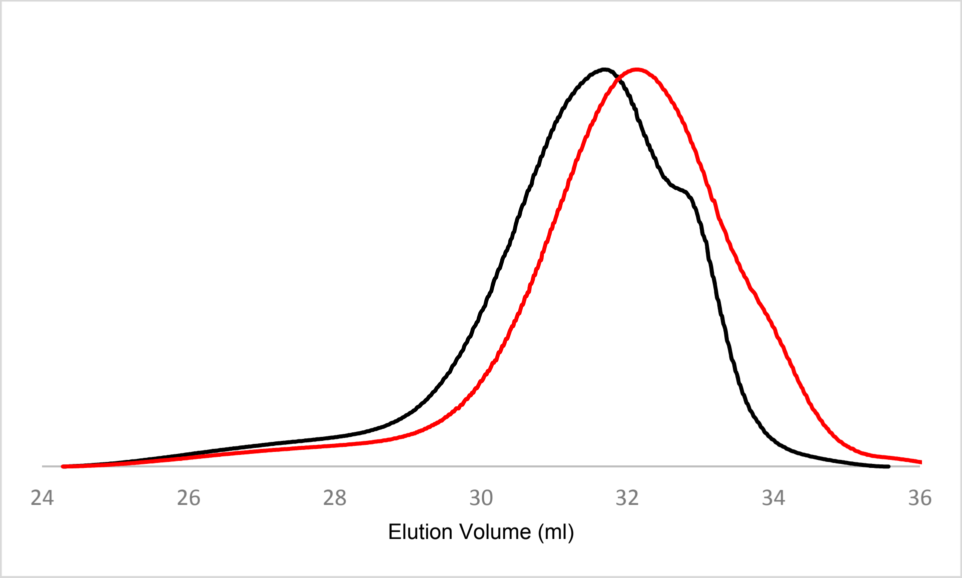

Figure S26. SEC elugram (eluent: THF) of P(PerPFP-*co*-PhMI) prepared in DCE with a feed ratio of 1:2 of PerPFP and PhMI before (black) and after (red) functionalization with *n*-benzylamine.
